# A fully iPS-cell-derived 3D model of the human blood–brain barrier for exploring neurovascular disease mechanisms and therapeutic interventions

**DOI:** 10.1038/s41593-025-02123-w

**Published:** 2025-12-15

**Authors:** Judit González-Gallego, Katalin Todorov-Völgyi, Stephan A. Müller, Sophie Antesberger, Mihail Ivilinov Todorov, Rainer Malik, Rita Grimalt-Mirada, Carolina Cardoso Gonçalves, Martina Schifferer, Georg Kislinger, Isabel Weisheit, Barbara Lindner, Dennis Crusius, Joseph Kroeger, Mila Borri, Ali Erturk, Mark Nelson, Thomas Misgeld, Stefan F. Lichtenthaler, Martin Dichgans, Dominik Paquet

**Affiliations:** 1 Institute for Stroke and Dementia Research (ISD), University Hospital, LMU Munich, Munich, Germany; 2 Graduate School of Systemic Neuroscience (GSN), LMU Munich, Munich, Germany; 3 German Center for Neurodegenerative Diseases (DZNE) Munich, Munich, Germany; 4 Neuroproteomics, School of Medicine and Health, Klinikum rechts der Isar, Technical University of Munich, Munich, Germany; 5 Institute for Tissue Engineering and Regenerative Medicine (iTERM), Helmholtz Center Munich, Neuherberg, Germany; 6 Munich Cluster for Systems Neurology (SyNergy), Munich, Germany; 7 Institute of Neuronal Cell Biology, Technical University of Munich, Munich, Germany; 8 Institute for Intelligent Biotechnologies (iBIO), Helmholtz Center Munich, Neuherberg, Germany; 9 Deep Piction, Munich, Germany; 10 School of Medicine, Koç University, Istanbul, Turkey; 11 Department of Pharmacology, University of Vermont, Burlington, VT USA; 12 Division of Cardiovascular Sciences, University of Manchester, Manchester, UK; 13 German Centre for Cardiovascular Research (DZHK), Munich, Germany

**Keywords:** Blood-brain barrier, Experimental models of disease, Stroke, Stem-cell differentiation

## Abstract

Blood–brain barrier (BBB) integrity is critical for brain homeostasis, with malfunctions contributing to neurovascular and neurodegenerative disorders. Mechanistic studies on BBB function have been mostly conducted in rodent and in vitro models, which recapitulate some disease features, but have limited translatability to humans and pose challenges for drug discovery. Here we report on a fully human induced pluripotent stem (iPS)-cell-derived, microfluidic three-dimensional (3D) BBB model consisting of endothelial cells (ECs), mural cells and astrocytes. Our model expresses typical fate markers, forms a barrier in vessel-like tubes and enables perfusion, including with human blood. Deletion of *FOXF2* in ECs, a major risk gene for cerebral small vessel disease, induced key features of BBB dysfunction, including compromised cell junction integrity and enhanced caveolae formation. Proteomic analysis revealed dysregulated endocytosis and cell junction pathways. Disease features phenocopied those seen in mice with EC-specific Foxf2 deficiency. Moreover, lipid-nanoparticle-based treatment with *Foxf2* mRNA rescued BBB deficits, demonstrating the potential for drug development.

## Abstract (web summary)

Gonzalez-Gallego et al. developed a fully iPS-cell-based human three-dimensional blood–brain barrier (BBB) model and used it to study roles of the stroke risk gene *FOXF2* in BBB dysregulation, demonstrating its applicability for mechanistic research and drug development.

## Main

Proper function of the central nervous system requires a tightly controlled chemical and metabolic environment, which is regulated by the blood–brain barrier (BBB)^1^. The BBB is composed of brain endothelial cells (BECs), pericytes or smooth muscle cells and astrocytes^2,3^. BECs have unique characteristics that contribute to the formation of the BBB’s physical barrier, including low transcytosis rates and specialized tight and adherens junctions^3,4^.

Malfunction of the BBB critically contributes to both neurovascular and neurodegenerative disease^5^. Animal models have formed the backbone of research for studying the BBB in health and disease and investigating drug delivery approaches^6^. Although animal models have provided important insights into the physiology and functioning of the BBB, genetic and molecular differences limit translation of animal findings to humans, which may also have contributed to the failure of clinical trials based on drug candidates identified in mouse models of neurological conditions^6–8^. Hence, there is a medical need to develop models that recapitulate central aspects of the human BBB and its malfunction in disease.

Recent advances in the use of induced pluripotent stem (iPS) cells for translational research^9^ and differentiation protocols for BBB cell types^10^ allow generation of human in vitro models. Most current BBB models are transwell-based co-cultures with ECs and astrocytes or mural cells, or their combination, cultured on different sides of a semipermeable membrane. Although these models have shown phenotypes closer to the in vivo condition than monocultures^11^, they still represent a simplification. Moreover, BECs in vivo usually reside in perfused tubular structures and respond to changes in dimensionality by altering gene expression and activating different signaling pathways, indicating the importance of developing more physiological, vessel-like three-dimensional (3D) models^12^. Recently, microfluidic systems combining primary and iPS-cell-derived cells have provided a promising alternative for approximating the BBB^10,13–15^ and a BBB model derived exclusively from iPS cells has already been applied to study a neurodegenerative disease^16^. However, a well-characterized, fully iPS-cell-derived human in vitro model consisting of all BBB cell types, which recapitulates relevant aspects of human neurovascular diseases, has, to the best of our knowledge, not been described.

To develop a fully human microfluidic iPS-cell-derived multicellular model of the BBB we first established simple differentiation protocols of endothelial cells (iECs), pericytes (iPCs), smooth muscle cells (iSMCs) and astrocytes (iASs) from iPS cells. All cell types displayed high purity and yield and benchmarking by -omics to their primary human counterparts and published datasets validated our protocols. We then combined all BBB cell types in a microfluidic 3D model, which recapitulated key morphological and functional features of the BBB. To explore the potential of the new model for pathomechanistic studies, we examined deficiency of FOXF2, a transcription factor and recently identified risk gene for stroke and cerebral small vessel disease^17,18^. FOXF2 has been shown to regulate BEC–pericyte interactions, production of extracellular matrix and BBB formation^19–21^. Global inactivation of FOXF2 during embryonic development in mice recapitulates some phenotypes relevant for small vessel disease, such as BBB leakage and intracerebral hemorrhage^22^, establishing FOXF2 as a key player in BBB integrity. However, loss of FOXF2 in human model systems and its specific role in BECs has not been investigated. We therefore generated FOXF2-deficient iPS cells and—given the central role of ECs in BBB function—focused on studying FOXF2 loss in the ECs of our human BBB model. Indeed, we found that key features of BBB dysfunction and proteomic analysis revealed an impairment of pathways involved in cell adhesion and endocytosis, confirming a role of human FOXF2 in BBB regulation. The observed disease features phenocopy those seen in EC-specific *FOXF2*-deficient mice (*Foxf2*^*iECKO*^) developed in parallel, thus replicating key in vivo results in an accessible cell culture system and validating our iPS-cell-derived model to study BBB biology and malfunction.

Given the critical role of BECs in BBB function and their involvement in human disease, there is great interest in optimized delivery of therapeutics to BECs. Lipid nanoparticles (LNPs) carrying messenger RNA recently emerged as a therapeutic agent to treat infectious disease and cancer^23^ and efforts to optimize LNP selectivity for and delivery to BECs are ongoing^24^. To explore the applicability of our 3D BBB model for testing therapeutic LNP delivery, we treated our cultures with LNPs containing *Foxf2* mRNA, which largely rescued BBB impairment in *FOXF2* knockout cultures. Overall, our results establish our iPS-cell-derived 3D BBB model as a valuable resource for mechanistic and translational applications in neurovascular diseases.

## Results

### Differentiation of neurovascular cell types and proteomic characterization

**Fig. 1:**
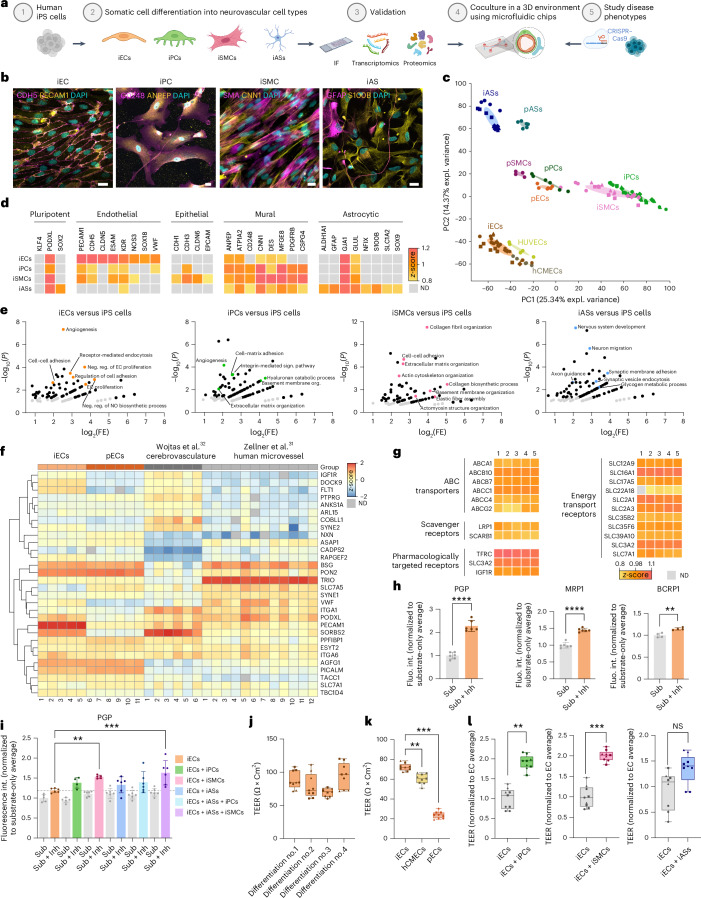
Optimized differentiation of iPS-cell-derived neurovascular cell types and deep characterization by proteomic analysis. **a**, Experimental outline: iPS cells (1) are differentiated into neurovascular cell types (2) and validated via IF, qPCR and proteomics (3). The 3D BBB model is generated by co-culturing differentiated cells in microfluid chips (4) and applied to study disease-associated mutations inserted by CRISPR–Cas9 genome editing at the iPS cell level (5). **b**, Representative IF of iECs, iPCs, iSMCs and iASs. Scale bar: 20 µm. **c**, PCA based on all proteins detected in iPS-cell-derived cells and human primary cells (*n* = 3–5 samples per group). **d**, Levels of cell-specific markers for iPS-cell-derived cells (*n* = 3–5 samples per group) in proteomics analysis shown by *z*-score. Gray squares represent proteins not detected (ND). **e**, PCA-based enrichment analysis of biological processes comparing iECs, iPCs, iSMCs and iASs with iPS cells. Proteins in black are significant (*P* < 0.05) and the ones marked in color are cell-type-specific processes (*P* < 0.05 and false discovery rate (FDR) < 0.05). **f**, Heatmap showing abundance of expression levels comparing iECs and pECs to published datasets^31,32^. **g**, Abundance of specific EC transporters and receptors (*n* = 5 samples per group). **h**, PGP, MRP1 and BCRP1 transporter function measured by fluorescent intensity (*n* = 4–6 replicates per group; normalized to substrate-only condition; comparison using two-tailed Student’s *t*-test, *P* < 0.0001, *P* = 0.002). **i**, PGP transporter function measured by fluorescent intensity in monocultures and co-cultures (*n* = 4–6 replicates per group; normalized to EC substrate-only condition; one-way analysis of variance (ANOVA) corrected for multiple comparisons by Tukey’s method, *P* = 0.0077, *P* = 0.0001). The dotted line shows average values for EC monoculture with substrate and inhibitor. **j**, TEER of iECs in four independent differentiations (*n* = 3 replicates per differentiation). **k**, TEER of iECs, hCMECs and pECs (*n* = 3 replicates per group; one-way ANOVA corrected for multiple comparisons by Tukey’s method, *P* = 0.005, *P* < 0.001). **l**, TEER of iECs in monoculture and co-culture (*n* = 3 replicates per group; normalized to iEC condition; Student’s *t*-test, *P* = 0.0072, *P* = 0.0007, *P* = 0.22). The TEER graphs show all three measurements (same symbol) from each replicate (different symbol) to indicate low variability. The bar graphs show mean ± s.d. The box and whisker plots in **j**–**l** show median, lower and upper quartiles and all data points. expl., explainable; FE, fold enrichment; Fluo. int., fluorescent intensity; Inh, inhibitor; NS, not significant; org., organization; sign., signaling; Sub, substrate. Illustrations in **a** created with BioRender.com. Source data

**Extended Data Fig. 1:**
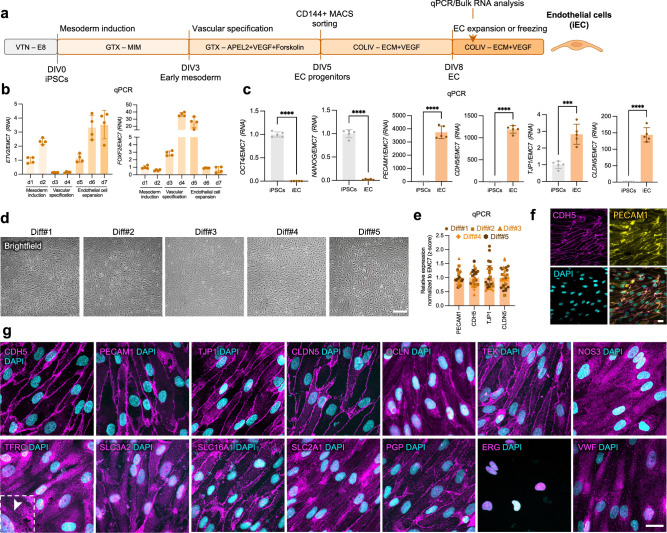
Differentiation and characterization of iPSC-derived endothelial cells (iEC). **a**. Overview of iEC differentiation. **b**. Relative RNA abundance of *ETV2* and *FOXF2* during iEC differentiation measured by qPCR (n = 4 replicates/day). **c**. Relative RNA abundance of pluripotency and EC markers in iEC and iPSCs (n = 5 replicates/group; normalized to iPSCs; two-tailed T-test, P < 0,0001, P = 0,0002) measured by qPCR. **d**. Brightfield images of 5 independent endothelial cell differentiations. Scale bar 25 µm. **e**. Relative RNA abundance of EC-markers in 5 independent differentiations measured by qPCR (n = 4-6 replicates/differentiation). Samples are normalized to average of each sample (z-score) to illustrate variation between samples. **f**. Individual channels of representative IF from Fig. 1b. Scale bar 20 µm. **g**. Representative IF of iEC stained for EC-markers. Scale bar 25 µm. Bar graphs show mean ± SD. Illustration in **a** created with BioRender.com. Source data

**Extended Data Fig. 2:**
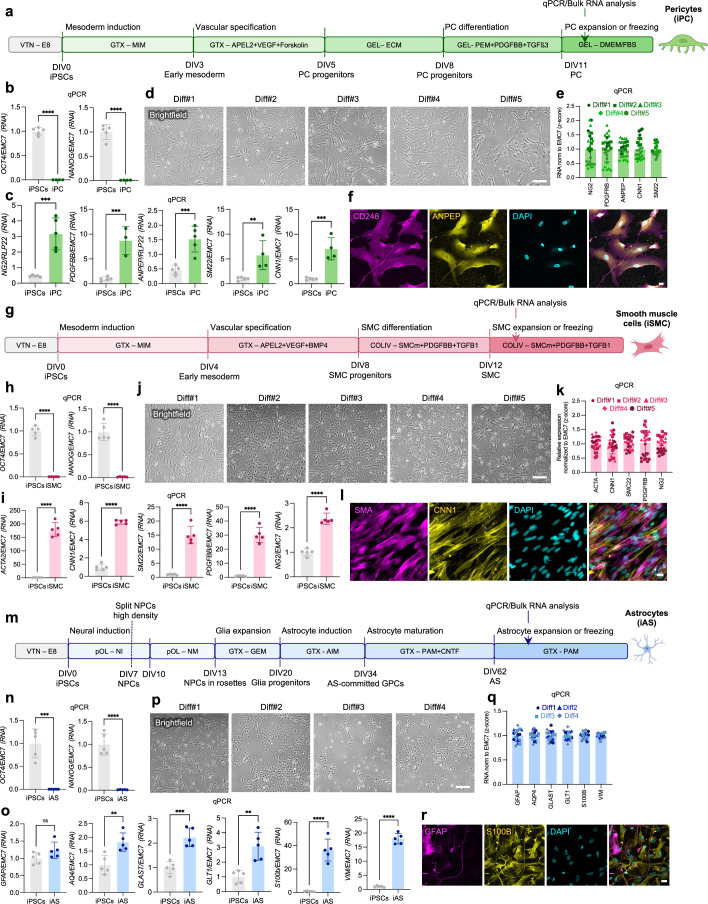
Differentiation and characterization of iPSC-derived mural cells (iPC and iSMC) and astrocytes (iAS). **a**. Overview of iPC differentiation. **b-c**. Relative RNA abundance of pluripotency markers (**b**) and mural cell-specific cell markers (**c**) in iPC and iPSCs measured by qPCR (n = 3-5 replicates/group; normalized to iPSCs; two-tailed T-test, P < 0,001, P=0.078). **d**. Brightfield images of 5 independent pericyte differentiations. Scale bar 25 µm. **e**. Relative RNA abundance of mural-markers in 5 independent differentiations measured by qPCR (n = 4-6 replicates/differentiation). Samples are normalized to average of each sample (z-score) to illustrate variation between samples. **f**. Individual channels of representative IF from Fig. 1b. Scale bar 20 µm. **g**. Overview of iSMC differentiation. **h-i**. Relative RNA abundance of pluripotency markers and mural cell-specific cell markers in iSMC and iPSCs measured by qPCR (n = 5 replicates/group; normalized to iPSCs; two-tailed T-test, P < 0,0001). **j**. Brightfield images of 5 independent smooth muscle cell differentiations. Scale bar 25 µm. **k**. Relative RNA abundance of mural-markers in 5 independent differentiations measured by qPCR (n = 5-6 replicates/differentiation). Samples are normalized to average of each sample (z-score) to illustrate variation between samples. **l**. Individual channels of representative IF from Fig. 1b. Scale bar 20 µm. **m**. Overview of iAS differentiation. **n-o**. Relative RNA abundance of pluripotency markers (**n**) and astrocytic-specific cell markers (**o**) in iAS and iPSCs measured by qPCR (n = 5 replicates/group; normalized to iPSCs; two-tailed T-test, P < 0,001, P < 0,0001, P = 0,1058, P = 0,0045, P = 0,0021). **p**. Brightfield images of 5 independent endothelial cell differentiations. Scale bar 25 µm. **q**. Relative RNA abundance of AS-markers in 4 independent differentiations measured by qPCR (n =  4 replicates/differentiation). Samples are normalized to average of each sample (z-score) to illustrate variation between samples. **r**. Individual channels of representative IF from Fig. 1b. Scale bar 20 µm. Bar graphs show mean ± SD. Illustrations in **a**, **g** and **m** created with BioRender.com. Source data

We first established, optimized and validated somatic cell differentiation protocols of iPS cells into all BBB-forming cell types (Fig. 1a). The iECs were differentiated by mesoderm induction and vascular specification. On day 5 in vitro (DIV5), iECs were selected by CDH5 (CD144) labeling and magnetic activated cell sorting (MACS) and subsequently cultured in EC medium for up to five passages (Extended Data Fig. 1a). Before purification, iECs already showed an upregulation of the early endothelium-specific transcription factor *ETV2* and *FOXF2*, which is enriched in BECs (Extended Data Fig. 1b). The iECs downregulated the pluripotency markers *OCT4* and *NANOG* and, after CDH5 selection and propagation, showed an enrichment of key endothelial transcripts such as *PECAM1*, *CDH5*, *CLDN5* and *TJP1* (Extended Data Fig. 1c). Terminally differentiated iECs reproducibly displayed typical morphologies and expressed endothelial markers at the mRNA level (Extended Data Fig. 1d,e). They also formed typical adherens junctions containing PECAM1 and CDH5 protein (Fig. 1b and Extended Data Fig. 1f) and expressed a variety of other typical brain endothelial markers at the expected subcellular locations in immunofluorescence (IF) staining (Extended Data Fig. 1g). The iPCs were differentiated together with iECs up to DIV5. The CDH5-negative MACS fraction was further used to differentiate iPCs as previously described with some modifications^25^ (Extended Data Fig. 2a). The iPCs downregulated pluripotency markers and upregulated transcripts characteristic for mural cells, such as *SM22* (*TAGLN*) and *CNN1* (Extended Data Fig. 2b,c). Terminally differentiated iPCs reproducibly displayed typical morphologies and expressed pericyte markers at the mRNA and protein levels, such as ANPEP and CD248 (Fig. 1b and Extended Data Fig. 2d–f). The iPS-cell-derived smooth muscle cells (iSMCs) were differentiated by mesoderm induction and further specification by culture in SMC medium (Extended Data Fig. 2g). The iSMCs downregulated pluripotency markers and upregulated mural-cell-specific transcripts such as *SM22* and *CNN1* (Extended Data Fig. 2h,i). At the stage used for the 3D BBB model, iSMCs reproducibly displayed typical morphologies and expressed SMC markers on mRNA and protein levels, such as SMA (Fig. 1b and Extended Data Fig. 2j–l).

**Extended Data Fig. 3:**
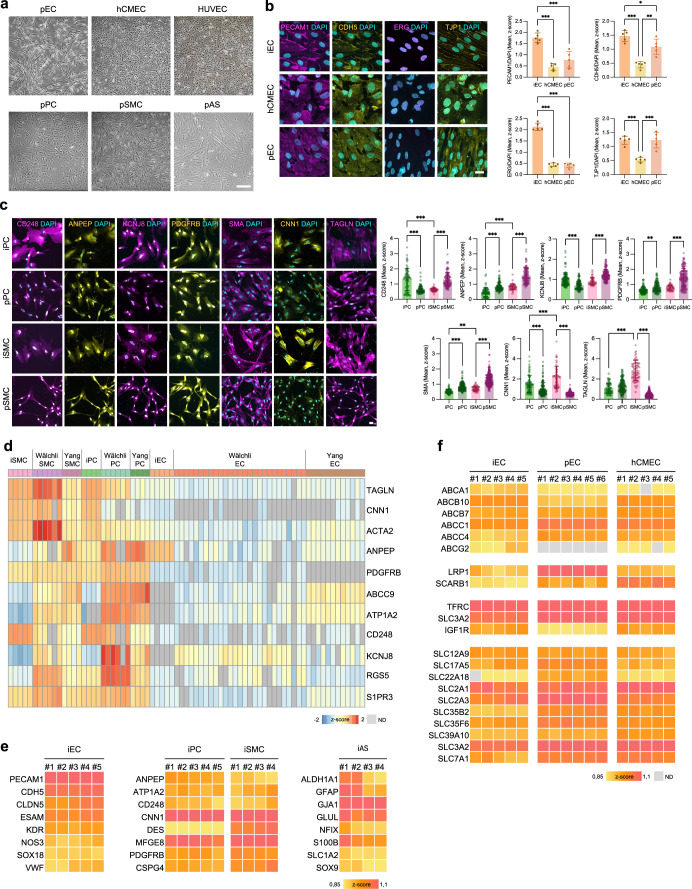
Culture and comparative characterization of primary cells. **a**. Brightfield images of pEC, hCMEC, HUVEC, pPC, pSMC and pAS. Scale bar 25 µm. **b**. Representative IF of iEC, hCMEC and pEC stained for EC-markers with corresponding quantification of mean intensity in each cell type normalized to DAPI count (n = 5 replicates/group; one-way ANOVA corrected for multiple comparisons by Tukey’s method, P < 0,001, P=0.001, P=0.03). Scale bar 25 µm. **c**. Representative IF of iPC, pPC, iSMC and pSMC for mural-cell markers with corresponding quantification of mean intensity in each cell type (n = 40-140 analyzed cells /group; one-way ANOVA corrected for multiple comparisons by Tukey’s method, P < 0,001, P=0.002) normalized to group average (z-score). Scale bar 25 µm. **d**. Abundance of mural-cell markers in bulk-RNA sequencing in iSMC, iPC and iEC in comparison to published studies^33,34^. **e**. Expression levels of cell-specific markers for iPSC-derived cells in 5 biological replicates in proteomics analysis shown as z-score. **f**. Expression levels of endothelial cell transporters in iEC, pEC and hCBMEC in biological (for iEC) or technical replicates (for pEC, hBMEC) (n = 5–6 replicates/group) normalized to group average (z-score). Grey squares represent not detected proteins (ND). Bar graphs show mean ± SD. Source data

The iASs were differentiated under serum-free conditions as previously described with some modifications^26^ (Extended Data Fig. 2m). They downregulated pluripotency markers and upregulated key astrocytic transcripts such as *GFAP*, *AQP4*, *GLAST*, *GLT1*, *VIM* and *S100B* (Extended Data Fig. 2n,o). Furthermore, terminally differentiated iASs reproducibly displayed typical morphologies and expressed astroglial markers on mRNA and protein levels, such as S100B and glial fibrillary acidic protein (GFAP) (Fig. 1b and Extended Data Fig. 2p–r). To further validate our iPS-cell-derived cells, we obtained commercially available human primary cells and cell lines commonly used in the field^13,27–29^ for comparison: brain microvascular ECs (pECs and HBMECs), human umbilical vein endothelial cells (HUVECs), an immortalized BBB EC line (hCMECs and D3 cells), brain vascular pericytes (pPCs and HBVPs), brain vascular smooth muscle cells (pSMCs and HBVSMCs) and midbrain astrocytes (pASs and HAs). EC and mural cell types were first characterized morphologically and for expression of typical markers by IF and -omics analysis (see below) (Extended Data Fig. 3a–f). IF staining indicated that cell-type markers were expressed in iPS-cell-derived and primary cells at comparable levels. In mural cells, some markers described as enriched in pericytes were also enriched in iPCs compared to iSMCs (CD248, KCNJ8 and ABCC9), whereas the expression of others was similar in iPCs and iSMCs, which is consistent with our own and published RNA sequencing (RNA-seq) studies (Extended Data Fig. 3c,d).

**Extended Data Fig. 4:**
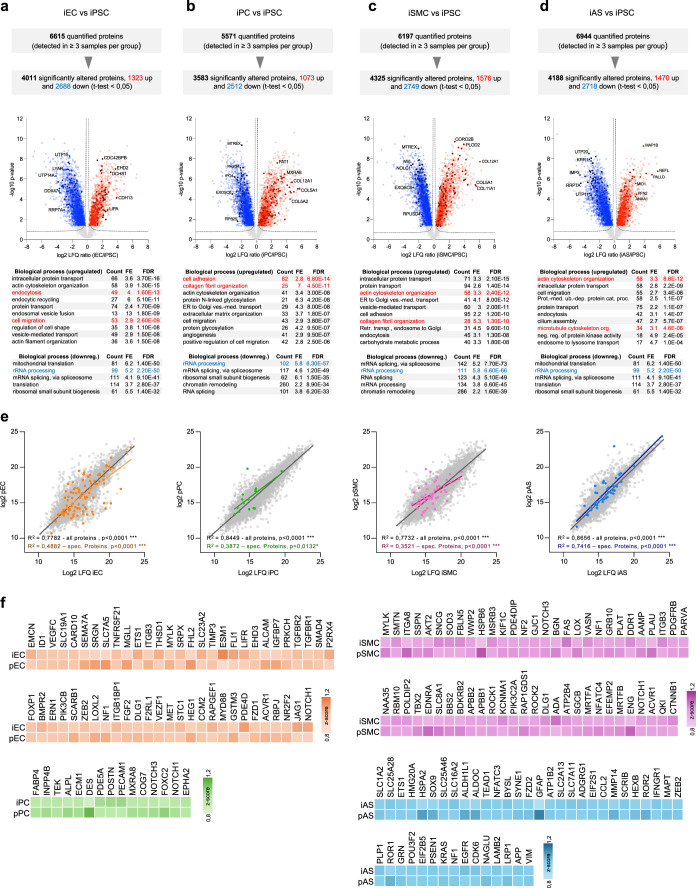
Characterization of iPSC-derived cells by proteomic analysis. **a-d**. Summary of LC-MS/MS and LFQ results (top), volcano plot of log_2_ LFQ ratio and -log_10_ p-values of all quantified proteins (middle), and enrichment analysis of biological processes of significantly dysregulated proteins in differentiated cells compared to iPSCs (bottom) for iEC (**a**), iPC (**b**), iSMC (**c**) and iAS (**d**). Red and blue circles indicate proteins that were significantly up- and downregulated respectively (n = 5 replicates/group, two-tailed t-test, p < 0,05). Dotted lines mark FDR threshold (p < 0,05). Proteins involved in colored enriched processes are marked with their gene name. Terms: Count: total enriched proteins, FE: fold enrichment, FDR: significance test of enriched terms. **e**. Correlation of expression levels of common (gray) and lineage-specific (colored) proteins between iEC vs pEC, iPC vs pPC, iSMC vs pSMC and iAS vs pAS. Black or colored line marks simple linear regression. **f**. Abundance of all lineage-specific proteins in iEC and pEC, iPC and pPC, iSMC and pSMC, iAS and pAS (n = 5 replicates/group). Bar graphs show mean ± SD.

**Extended Data Fig. 5:**
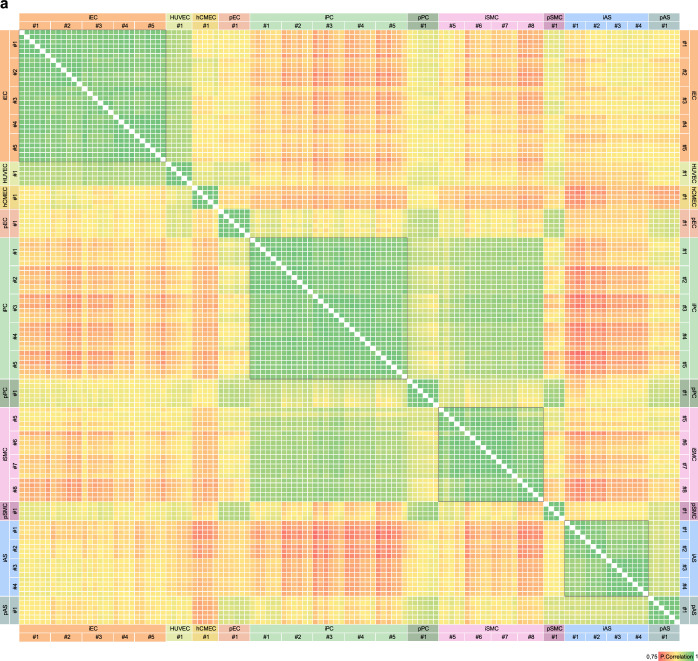
Pearson correlation of biological and technical replicates of iPSC-differentiated and primary cells. **a**, Heatmap indicating Pearson correlation of log2 transformed protein LFQ intensities between all samples analyzed by proteomics. Biological replicates are numbered (#1 etc.) and contain 4–5 technical replicates each, which are displayed as single boxes color-coded for Pearson value. Note very high correlation between both biological and technical replicates within the same cell type (surrounded by gray boxes), indicating high reproducibility within and across differentiations. Correlations are also high between iPSC-derived and primary cells of the same type, but lower between different cell types, as expected.

To compare iPS-cell-derived and primary cells, we performed quantitative label-free proteomics (Extended Data Fig. 4a–d and Supplementary Table 1): Unsupervised principal component analysis (PCA) revealed that iECs clustered with hCMEC BECs and close to HUVECs, whereas pECs clustered closer to the other primary cell types (Fig. 1c). The iPS-cell-derived (iPCs) mural cells (iSMCs) clustered closely with primary mural cells, albeit without a clear separation into different types of mural cells in both groups, indicating overall high similarity (‘Discussion’). The iASs clustered close to pASs and far from ECs, discriminating this cell type from the mesoderm-derived cells. To check for reproducibility of our differentiations within an experiment and across multiple experiments, we performed correlation analysis between iPS-cell-derived and primary somatic cell types, which confirmed consistently high correlations between samples of the same cell type (Extended Data Fig. 5a).

**Fig. 2:**
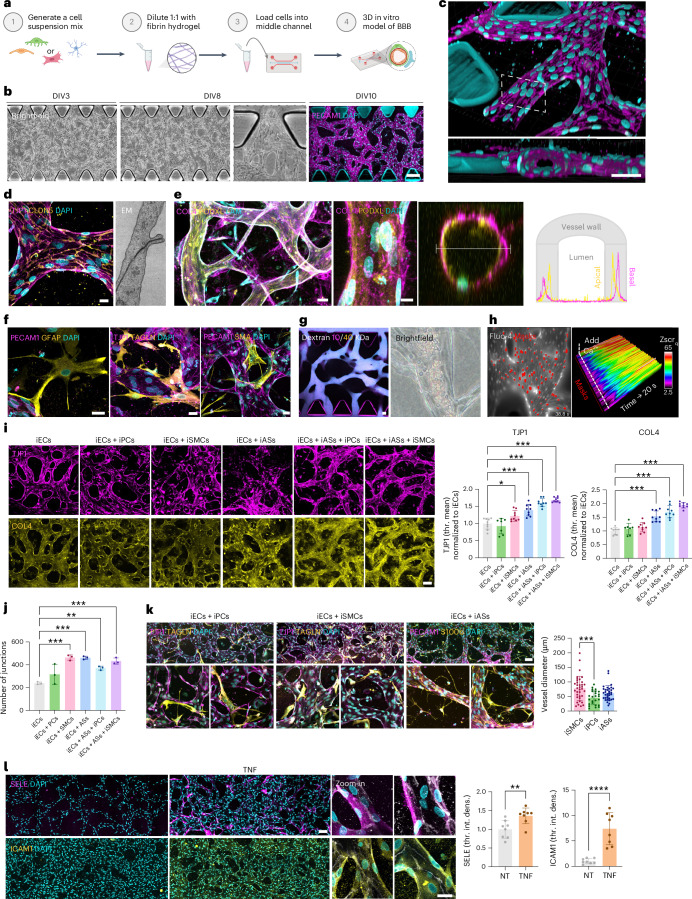
Generation and characterization of a fully iPS-cell-derived human 3D BBB model to investigate neurovascular disorders. **a**, Experimental outline. **b**, Representative images of vessel-like tubes in the microfluidic chamber at the indicated time points in brightfield or stained with PECAM1 and DAPI. Scale bar: 250 µm. **c**, The 3D rendering of **b**. Scale bar: 50 µm. **d**, Left: representative IF of ECs in the 3D BBB model expressing tight junction protein TJP1. Scale bar: 25 µm (left). Right: representative scanning electron microscope (SEM) image of endothelial junction. **e**, Left: representative IF for extracellular matrix and glycocalyx proteins. Scale bar: 50 µm. Middle and right: vessel side view showing apical-basal polarity marker expression (middle) and vessel cross-section (right) with intensity profiles of COL4 and PODXL staining at the indicated line. **f**, Representative IF of iAS (left, GFAP), iPC (middle, TAGLN) and iSMC (right, SMA) localization in 3D BBB culture stained with either PECAM1 or TJP1. Scale bar: 25 µm. **g**, Left: maximum intensity projection view of 3D BBB model perfused with 10-kDa and 40-kDa dextran. Scale bar: 50 µm. Right: 3D BBB model perfused with human blood. **h**, Calcium waves in iECs loaded with Fluo-4 in the 3D BBB model after calcium addition. Imaged masks are indicated in red (left). The signal strength is indicated as *Z*scr_q_ (Supplementary Video 2). **i**, Representative IF of 3D monocultures and co-cultures stained for TJP1 or COL4 with quantification of mean intensity (relative to iEC monoculture) (*n* = 7–9 replicates per group; normalized to iEC condition; one-way ANOVA corrected for multiple comparisons by Tukey’s method, *P* = 0.02, *P* < 0.001). Scale bar: 100 µm. **j**, Quantification of junctions of monocultures and co-cultures (Methods; *n* = 3 replicates per group; normalized to iEC condition; one-way ANOVA corrected for multiple comparisons by Tukey’s method, *P* < 0.001, P = 0.007). **k**, Representative IF of 3D co-cultures stained for markers of iECs (TJP1 or PECAM1), iASs (S100b) and iPCs or iSMCs (TAGLN). Left: iECs + iPCs; middle: iECs + iSMCs; right: iECs + iASs. The diameter of the vessels next to touching cells is quantified (*n* = 25–36 replicates per group; one-way ANOVA corrected for multiple comparisons by Tukey’s method, *P* < 0.001). Scale bar: 100 µm, for overview images, and 25 µm, for zoom images. **l**, Representative IF of 3D model stained for E-selectin and ICAM-1 nontreated (left) or after TNF treatment (middle), with respective quantification of mean intensity (right, *n* = 8 replicates per group; normalized to the NT condition; two-tailed Student’s *t*-test, *P* = 0.0061, *P* < 0.0001). Scale bar: 100 µm for overview images and 50 µm for zoom images. The bar graphs show mean ± s.d. DIV, division; int. dens., integrated density; thr., threshold. Illustrations in **a** created with BioRender.com. Source data

Parsing the proteomics profiles for specific markers, we found that pluripotency markers were either no longer detected in the differentiated cells, such as KLF4, or at very low levels, like SOX2 or PODXL, which is also present in the glycocalyx of vascular cells (Figs. 1d and 2e). The iECs significantly upregulated key endothelial markers like PECAM1, CDH5, CLDN5, ESAM, KDR (FLT1), NOS3, SOX18 and VWF. Intriguingly, expression of these typical marker genes in iECs was comparable or higher than in primary BECs (Extended Data Fig. 3b,e). Concomitantly, we could exclude aberrant differentiation into cells of epithelial identity, which has been observed in cells obtained from a broadly used protocol aiming for EC differentiation^30^, because our iECs did not express epithelial markers such as CDH1, CDH3, CLDN6 or EPCAM (Fig. 1d). The iPCs and iSMCs significantly upregulated typical mural cell markers, such as ANPEP, ATP1A2, CNN1 and CSPG4 (NG2), with higher expression of subtype-specific markers, such as ANPEP for iPCs and DESM for iSMCs, in the respective cell types (Fig. 1d). The iASs significantly upregulated expected astrocytic markers such as ALDH1A1, GFAP, GJA1, GLUL, NFIX, S100B, SLC1A2 and SOX9. All cell types showed high reproducibility in independent differentiations (Extended Data Fig. 3e). The comparative proteomic profiles of all cell types and iPS cell differentiation protocols are accessible through our interactive NeuroVasP database for NeuroVascular cell Proteins, available at https://dbNeuroVasP.isd-muc.de.

To further validate our differentiations at the level of active biological processes, we performed enrichment analysis for gene ontology (GO) biological processes based on PCA for each specific cell type versus iPS cells (Fig. 1e). The iEC proteome was enriched for EC-typical biological processes, such as angiogenesis, EC proliferation, nitric oxide biosynthesis and endocytosis. Analysis of mural cells also yielded expected biological processes, such as cell–matrix adhesion, basement membrane organization and collagen fibril organization, whereas iASs enriched AS-typical processes, including nervous system development, glycogen metabolic processes and axon guidance. In addition, we performed a broader biological processes enrichment analysis based on significantly upregulated and downregulated proteins in our derived cells versus iPS cells (Extended Data Fig. 4a–d and Supplementary Table 2), which showed similar cell-specific processes to be enriched in comparison to analysis based on top hits found by PCA, such as endocytosis for iECs, collagen fibril organization for mural cells and microtubule cytoskeleton organization for iASs. Processes associated with DNA and RNA metabolism were enriched in downregulated proteins in all cell types. Last, comparison of protein expression levels between our iPS-cell-derived neurovascular cell types and their corresponding primary cells revealed a strong correlation for all common proteins, as well as for lineage-specific proteins, suggesting similarity of proteomes not only at the qualitative but also at the quantitative level (Extended Data Fig. 4e,f).

**Extended Data Fig. 6:**
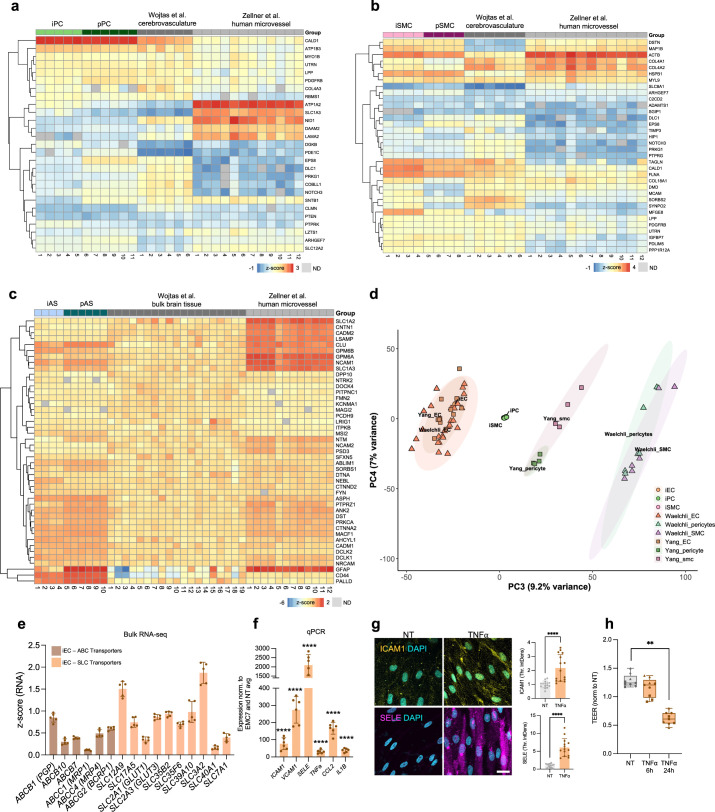
Characterization of iPSC-derived cells by comparison with published datasets, and functional characterization for transporter expression and TEER in iECs. **a-c**. Heatmap showing abundance of expression levels comparing iPC-pPC (**a**), iSMC-pSMC (**b**) and iAS-pAS (**c**) to published datasets^31,32^. **d**. PCA based on all transcripts detected in iEC, iPC and iSMC compared to published datasets^33,34^. EC subtypes present in the published data have been grouped into one general EC group for simplification. **e**. Relative RNA abundance of ABC and SLC transporters in iEC measured by bulk-RNA sequencing (n = 5 replicates) normalized to whole group average (z-score) to show relative expression. **f**. Relative RNA expression of inflammation markers in iEC after TNFα treatment normalized to EMC7 (n = 6 replicates/sample; normalized to NT condition; two-tailed T-test, P < 0,0001). **g**. Representative immunohistochemistry of iEC after non-treated (NT) and TNFα treatment stained for ICAM1 and E-selectin with the corresponding quantification of mean intensity (n = 15 replicates/sample; normalized to NT condition; two-tailed T-test, P < 0,0001). **h**. TEER of iEC after TNFα treatment (relative to NT condition) (n = 3 replicates/sample; normalized to NT condition; one-way ANOVA corrected for multiple comparisons by Tukey’s method, P = 0,001). The TEER graphs show all three measurements (same symbol) from each replicate (different symbol) to indicate low variability. Bar graphs show mean ± SD. Box and whisker plots show median, lower and upper quartile and all data points. Source data

In addition to comparing iPS-cell-derived and primary cells, we validated our proteomic data by comparing both to two different published datasets of human brain vascular cells. We used an enriched microvessel dataset from ref. ^31^, as well as two datasets from ref. ^32^ containing either vascular cells in the cerebrovascular fraction (to compare to iECs and mural cells), or astrocytes in the whole-brain preparation (to compare to iASs). As these datasets contained proteomic data from multicellular tissues, we first enriched for cell-type-specific proteins based on a single-cell RNA-seq (scRNA-seq) study^33^ (Methods). We then compared these cell-type-enriched data to our proteomic data from iPS-cell-derived and primary single-cell types. Intriguingly, cell-specific markers showed largely comparable protein expression levels across our iPS-cell-derived cells, primary cells and vascular tissue isolated from human samples. In most cases, similarities between iPS-cell-derived and in vivo datasets were as high or even higher than those between the two in vivo datasets (Fig. 1f and Extended Data Fig. 6a–c). For additional validation, we performed bulk RNA-seq followed by PCA for our endothelial and mural cell differentiations compared to two published scRNA-seq studies^33,34^. We focused on comparison of principal components PC3 and PC4, because PC1 and PC2 separated individual datasets, but not cell types from each other (Methods). The iECs clustered closely with ECs from both studies, whereas iPS-cell-derived mural cells clustered closer to the dataset from ref. ^33^ than to that from ref. ^34^ and the two in vivo datasets to each other, thus largely corroborating our proteomic validation (Extended Data Fig. 6d and Supplementary Table 4).

As a last aspect, we investigated whether our differentiated cells also acquire classic functional properties of BBB cells. ECs in the BBB typically express transporters regulating passage of cargo^1^ and we found key proteins such as ABC transporters (for example, ABCC1/MRP1, ABCG2/BCRP1 and ABCA1), scavenger receptors (LRP1 and SCARB1) and glucose and monocarboxylate transporters (SLC2A1, SLC16A1 and SLC7A1) to be reproducibly expressed^35^ at comparable levels to primary BECs in our proteomics dataset (Fig. 1g and Extended Data Fig. 3f), which we confirmed for several markers by IF staining and bulk RNA-seq (Extended Data Figs. 1g and 6e). Proteins relevant for translational and pharmacological studies^36–38^, such as transferrin receptor (TFRC), SLC3A2 (CD98h) and IGF1R, were also reproducibly present, indicating the potential to study pharmacological interventions based on these receptors in vitro. To study functionality of these transporters, we measured their activity before and after specific inhibition using established transwell assays^39,40^, which confirmed transporter activity for PGP, MRP1 and BCRP1 (Fig. 1h). Co-culture of iECs and iSMCs, but especially triple culture of iECs, iSMCs and iASs, further increased transport activity (Fig. 1i), indicating beneficial effects of the more BBB-like co-culture. Similar experiments with iPCs followed the same trend, but did not reach significance.

As another functional paradigm, we determined transendothelial electrical resistance (TEER) as a measure for barrier properties. Our iECs reproducibly reached TEER values of around 70–100 Ω cm^2^ (Fig. 1j), which was significantly higher than those of hCMECs or pECs (Fig. 1k), and comparable to or higher than in previous studies (Supplementary Table 3) with iECs, albeit still lower than resistances described for the BBB in vivo^41^. Intriguingly, co-culture with both mural cell types significantly increased TEER (Fig. 1l) compared to monoculture, in line with previous reports, whereas iASs showed only a trend in that direction. As another functional aspect, we also tested whether iECs can be stimulated using inflammatory cytokines, an important endothelial function with disease relevance^13,29^. Indeed, treatment with tumor necrosis factor (TNF) increased the expression of several inflammation markers, such as intercellular adhesion molecule 1 (ICAM-1) and E-selectin, at RNA and protein levels, and lowered TEER values (Extended Data Fig. 6f–h).

In conclusion, our optimized iPS cell differentiation protocols yield cell types that express key cell-specific BBB markers with an overall high similarity to corresponding human cell types in the brain and display functional features typical for BBB-like cells.

### Characterization of a fully iPS-cell-derived human 3D BBB model

To generate a fully iPS-cell-derived 3D in vitro model of the BBB, we adapted a previously described protocol for co-culturing cells in 3D microfluidic chips^13^. Fully differentiated iECs and iASs were combined in a fibrin hydrogel at defined ratios, together with either iSMCs or iPCs to model cerebral vessels surrounded by either mural cell type and injected into the middle channel of a microfluidic chip (Fig. 2a). Cultures were fed every 24 h by gravity-driven flow, adding different volumes of vascular (VASC) medium in the left and right ports, to promote endothelial network formation. Then, 3 d after embedding, iECs self-organized into premature vascular networks and continued their sprouting over the following week, when branched vessel-like tubes were observed (Fig. 2b), which persisted for over a week.

Co-culture of iECs with iASs and either iPCs or iSMCs resulted not only in ECs forming branched vessel-like structures shown by PECAM1 expression, but also the arrangement of cells to form a vessel lumen (Fig. 2c). The iECs expressed tight junction markers such as TJP1 and CLDN5 and presented electron-dense areas between adjacent cells shown by electron microscopy (EM) (Fig. 2d). After 7 d, a rich extracellular matrix containing collagen (COL4) and laminin (LAM) was observed. ECs showed organized polarity with secretion of podocalyxin (PODXL) toward the luminal or apical side and of collagen toward the abluminal or basal side (Fig. 2e). The iASs remained star shaped and extended their processes toward the endothelium. TAGLN-positive iPCs were positioned along the abluminal surface of ECs. Alternatively used iSMCs were positioned between endothelial branching points and expressed markers such as SMA (Fig. 2f). The lumina of the vessel-like tubes could be perfused with 10-kDa and 40-kDa dextran and human blood (Fig. 2g and Supplementary Video 1) under gravity flow without leakage, demonstrating the functionality of ECs in forming a functional barrier. BECs exhibit calcium signals that reflect calcium release through IP3R channels^42^. To examine the capacity of ECs to propagate calcium, we applied calcium to the medium and saw slow transient calcium waves traveling across the cell layer in a coordinated manner, suggesting electrical coupling (Fig. 2h and Supplementary Video 2). To explore the beneficial effects of co-culture of iECs with mural cells and/or astrocytes in three dimensions, we quantified formation of tight junctions (based on TJP1 expression) and extracellular matrix (based on COL4 expression) and found that addition of astrocytes alone, but especially together with mural cells, increased both parameters, resulting in a more BBB-like phenotype (Fig. 2i). Quantification of branching further showed a significant increase in the co-cultures compared to monoculture samples, suggesting promotion of vessel complexity by other BBB cells. (Fig. 2j). Furthermore, as in the brain, the diameter of vessels associated with iPCs was smaller than that with iSMCs (Fig. 2k). Last, perfusion of vessels with TNF-containing medium induced an inflammatory phenotype with upregulation of E-selectin and ICAM-1, indicating the potential of the model to investigate vascular inflammatory processes (Fig. 2l). Taken together, we engineered a fully iPS cell-based, 3D human brain vessel model recapitulating key features of the BBB, such as formation of perfusable tubes with apical-basal topology, extracellular matrix generation, barrier formation and representation of typical cell–cell interactions and functions.

### FOXF2 deficiency dysregulates caveolae and cell junctions in the 3D BBB model, phenocopied in vivo

**Fig. 3:**
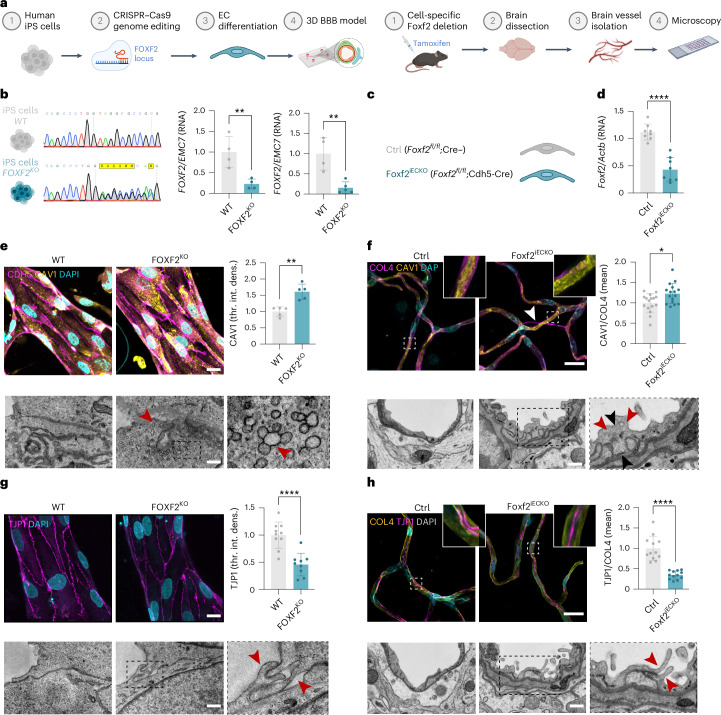
FOXF2 deficiency dysregulates caveolae and cell junction formation in a human 3D BBB model, which is phenocopied in vivo*.* **a**, Experimental overview for generating human FOXF2^KO^ iPS cells and EC-specific *FOXF2*^KO^ mice. **b**, Sequencing of the *FOXF2* KO iPS cells and qPCR measurement for *FOXF2* in iPS cells (middle) and iECs (right) (*n* = 4–5 replicates per group, normalized to EMC7 and WT, respectively; two-tailed Student’s *t*-test, *P* = 0.0079, *P* = 0.0033). **c**, Overview of *Foxf2*^iECKO^ and control (Ctrl) mouse lines. **d**, Relative RNA levels of *Foxf2* in full tissue measured by qPCR (*n* = 8 replicates per group, normalized to *Actb* and Ctrl, respectively; two-tailed Student’s *t*-test, *P* < 0.0001). **e**, Top: representative IF of the 3D BBB model stained for CDH5 and CAV1 with quantification of CAV1 mean intensity (*n* = 5 replicates per group; normalized to WT; two-tailed Student’s *t*-test, *P* = 0.001). Left: WT; middle: FOXF2^KO^. Scale bar: 25 µm. Bottom: representative TEM of caveolae enrichment (marked with red arrows) in KO compared to WT (*n* = 1 replicate per group). Scale bar: 0.5 µm. **f**, Top: representative IF of mouse brain-isolated vessels stained for CAV1 and COL4 with quantification of mean intensity of CAV1 normalized to COL4 (*n* = 16 replicates per sample; normalized to WT; two-tailed Student’s *t*-test, *P* = 0.0108). Left: control; middle: Foxf2^i^^ECKO^. Scale bar: 20 µm. The arrow shows a string vessel. Bottom: representative TEM image of endothelium thickening (black arrows) and caveolae enrichment (red arrows) in Foxf2^iECKO^ compared to the Ctrl vessel (*n* = 1 replicate per sample). Scale bar: 1 µm. **g**, Top: representative IF of the 3D BBB model stained for TJP1 with quantification of TJP1 integrated intensity (*n* = 9–10 replicates per group; normalized to WT; two-tailed Student’s *t*-test, *P* < 0.0001). Left: WT; middle: FOXF2^KO^. Scale bar: 25 µm. Bottom: representative TEM image of EC adhesion and tight junction regions in KO compared to WT (*n* = 1 replicate per group), with opening (red arrow) on the right. Scale bar: 0.5 µm. **h**, Top: representative IF of mouse brain-isolated vessels stained for TJP1 and COL4 with quantification of mean intensity of TJP1 normalized to COL4 protein expression (*n* = 12 replicates per sample; normalized to Ctrl; two-tailed Student’s *t*-test, *P* < 0.0001). Left: control; middle: Foxf2^iECKO^. Scale bar: 20 µm. Bottom: representative TEM image of endothelial tight junction protrusions (red arrow) in Foxf2^iECKO^ compared to Ctrl (*n* = 1 replicate per group), with enlarged area on the right. Scale bar: 1 µm. The bar graphs show mean ± s.d. Illustrations in **a**–**c** created with BioRender.com. Source data

**Extended Data Fig. 7:**
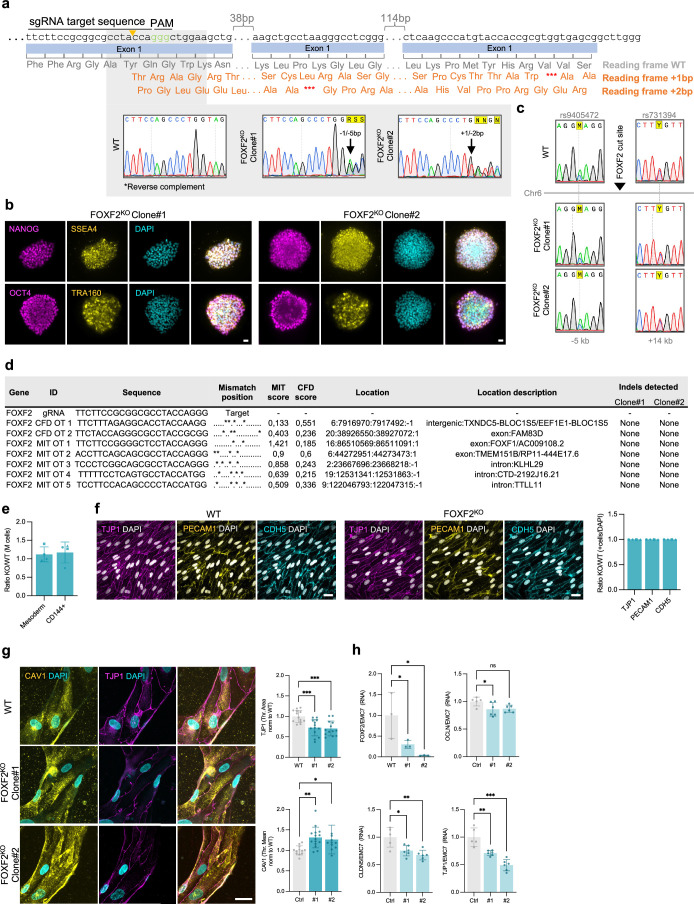
Generation of FOXF2^KO^ iPSCs by CRISPR/Cas9 mediated genome editing of human iPSCs and differentiation into iECs. **a**. *FOXF2* knockout strategy: Exon1 of *FOXF2* was targeted by a sgRNA (target and PAM sequence shown), generating a 1 base pair deletion on one allele combined with a 5 base pair deletion on the other for clone#1 and 1 base pair insertion on one allele combined with a 2 base pair deletion on the other for clone#2. The resulting frameshifts expose a nearby stop codon in both cases. **b**. Immunofluorescence analysis of undifferentiated-state markers: SSEA4, NANOG, TRA160 and OCT4 in FOXF2^KO^ lines. Scale bar 25 µm. **c**. Sanger sequences of unmodified heterozygous SNPs near the edited locus in WT and FOXF2^KO^ iPSC lines showing maintenance of both alleles and absence of on-target effects after editing. **d**. List of top five most similar off-target sites ranked by CFD and MIT prediction scores. Sites identified by both algorithms (CFD OT3, 4 and 5) are only shown once. No off-target effects were detected in both KO lines **e**. Yield of cells induced from iPSCs to mesoderm and expressing CD144 in the mesoderm fraction shown as WT/KO ratio (n = 4 differentiations). **f**. Representative IF of iEC stained for TJP1, PECAM1 and CDH5 in WT and FOXF2^KO^, quantified on the right (n = 4 differentiations). Scale bar 25 µm. **g**. Representative IF of two independent FOXF2^KO^ 3D cultures stained for CAV1 and TJP1 with quantification of mean intensity (n = 12–15 replicates/sample; normalized to WT; one-way ANOVA corrected for multiple comparisons by Tukey’s method, P < 0,001, P=0.0059, P=0.0335). **h**. Relative RNA abundance of *FOXF2*, *OCLN*, *CLDN5* and *TJP1* in two independent FOXF2^KO^ clones measured by qPCR (n = 3-6 replicates/sample; normalized to WT; two-tailed T-test, P=0.04, P=0.02, P=0.0475, P=0.0679, P=0.0101, P=0.0012, P=0.001, P < 0,0001). Bar graphs show mean ± SD. Source data

**Extended Data Fig. 8:**
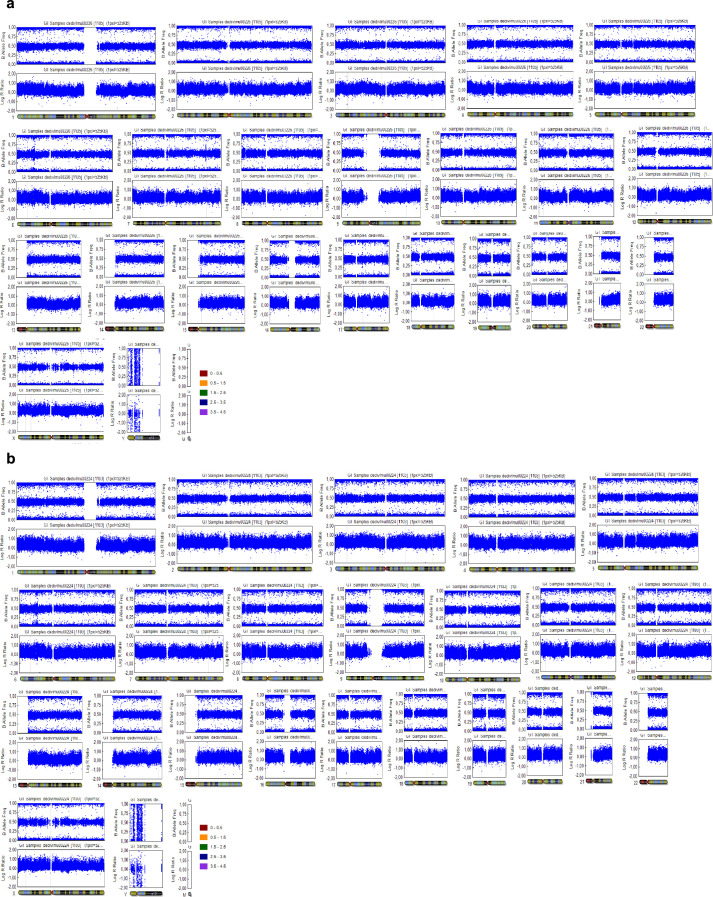
Karyotyping of FOXF2^KO^ edited iPSC lines. **a-b**. Genome-wide SNP analysis for molecular karyotyping for clone#1 (**a**) and clone#2 (**b**). All measured SNPs are indicated by blue dots. B allele frequencies (BAF) and Log R ratios are shown for each chromosome in the FOXF2^KO^ iPSC line. BAF values indicate normal zygosities on all chromosomes and Log R ratios show the absence of detectable insertions or deletions.

To explore the suitability of our model to investigate the role of FOXF2 in neurovascular dysfunction, identify cell-autonomous effects on ECs and crossvalidate our findings in an independent system, we deleted *FOXF2* in both human iPS cells (Fig. 3a,b) and ECs of adult mice (Fig. 3c,d). For the generation of FOXF2^KO^ iPS cells, we targeted the DNA-binding region in exon 1, following our previously described CRISPR genome-editing pipeline^43^. Genome editing led to a +1-bp or −5-bp insertion or deletion on each corresponding allele, exposing a premature stop codon. We also generated a second, independent knockout (KO) clone to validate key conclusions and rule out clone-specific effects (Fig. 3b and Extended Data Fig. 7a). FOXF2^KO^ iPS cell clone nos. 1 and 2 were characterized for undifferentiated state markers, absence of on-targets and off-targets and chromosomal integrity (Extended Data Figs. 7b–d and 8). As *FOXF2* is strongly expressed in ECs^33,44^, we further differentiated the iPS cells into iECs to study *FOXF2* deletion. As expected, FOXF2^KO^ iECs showed an almost complete downregulation of *FOXF2* RNA levels (Fig. 3b and Extended Data Fig. 7h), likely due to nonsense-mediated mRNA decay. To exclude effects of FOXF2 deficiency on overall endothelial differentiation, we confirmed that the differentiations yielded similar numbers of iECs which expressed typical markers (Extended Data Fig. 7e,f). To validate our findings from the human in vitro model, we used a mouse line with conditional endothelial (Cdh5-CreERT2; *Foxf2*^*fl/fl*^ or *Foxf2*^*iECKO*^) cell-specific inactivation of *Foxf2* (Fig. 3c), which was developed in a parallel in vivo study^45^. FOXF2 deficiency in these *Foxf2*^*iECKO*^ mice was confirmed by quantitative (q)PCR, compared to age-matched control mice (*Foxf2*^*fl/fl*^ or control) (Fig. 3d).

**Extended Data Fig. 9:**
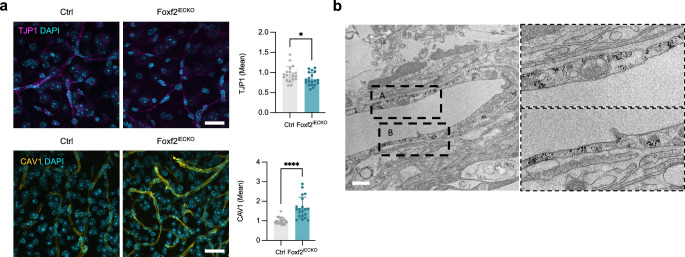
Caveolae enrichment and cell junction reduction induced by endothelial FOXF2 deficiency in the mouse. **a**. Representative immunohistochemistry of mouse brain slices stained for CAV1 (right) and TJP1 (left) with quantification of mean intensity (n = 19–20 replicates/sample; normalized to Ctrl; two-tailed T-test, P =0.038, P<0.0001. Scale bar 25 µm. **b**. Immunogold localization of CAV1 in KO endothelium using electron microscopy (n = 1 replicate/sample). Scale bar 0.5 µm. Bar graphs show mean ± SD. Source data

In vivo studies have shown that global *Foxf2* deletion leads to pronounced deficits in capillary ECs, including thickening, increased vesicular transport and longer tight junction surfaces^22^. Furthermore, two studies have described FOXF2 as a key player in ECs in the BBB^46,47^, but EC-specific loss of FOXF2 has not been studied. We therefore focused on analyzing whether cell-autonomous KO of *FOXF2* in human ECs is sufficient to cause BBB dysfunction. To assess whether our human 3D BBB model recapitulates phenotypes related to FOXF2 deficiency, we quantified the density of caveolae in iECs by IF and found CAV1 to be upregulated in 3D cultures containing FOXF2-deficient iECs combined with isogenic wild-type (WT) control iPCs and iASs, compared to cultures containing WT cells only. Correlative transmission electron microscopy (TEM) analysis confirmed caveolae to be enriched in ECs and, in addition, demonstrated morphological deficits evidenced by assemblies of multiple vesicular structures (Fig. 3e and Extended Data Fig. 7g). To independently validate these findings, we performed related experiments in *Foxf2*^*iECKO*^ mice and found that endothelial *Foxf2* deletion also led to an upregulation of CAV1 in isolated brain vessels (Fig. 3f) and mouse brain sections (Extended Data Fig. 9a). Similarly, correlative ultrastructure analysis revealed an endothelial thickening and vesicle enrichment (Fig. 3f). Immunogold labeling of mouse brain sections further revealed that most of these vesicles are Cav1-positive, suggesting an upregulation of caveolae-dependent endocytosis (Extended Data Fig. 9b).

We next turned our attention to barrier function and found that endothelial inactivation of FOXF2 led to downregulation of TJP1 in the human 3D BBB model (Fig. 3g and Extended Data Fig. 7g), thus phenocopying findings in mouse brain microvessels in vivo (Fig. 3h and Extended Data Fig. 9a). In line with the downregulation of TJP1, ultrastructural analysis revealed altered cell junctions with elongated protrusions and focal openings between the junctional membrane surfaces (Fig. 3g,h).

### Widespread barrier and cellular transport deficits in FOXF2-deficient iECs

**Fig. 4:**
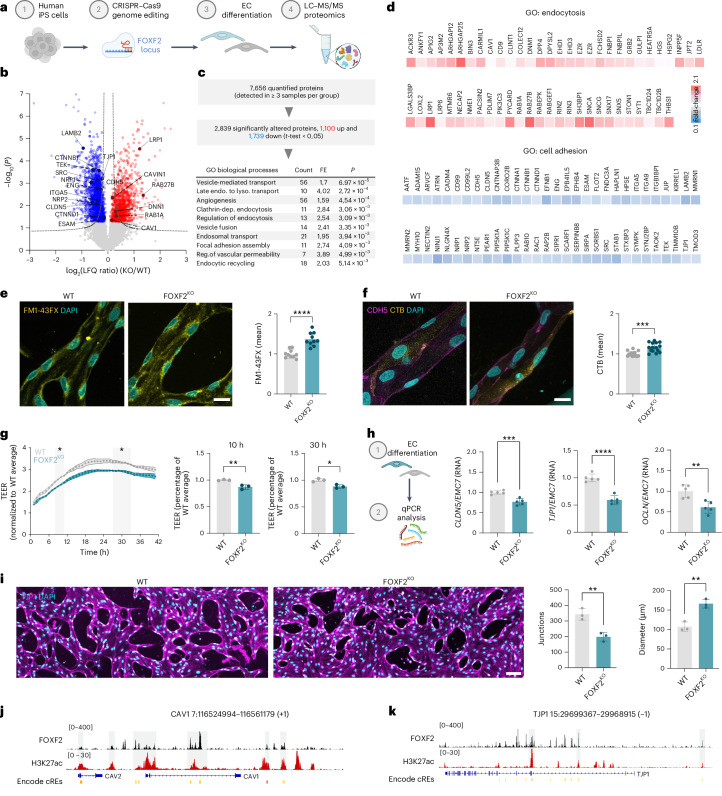
Proteomic and functional characterization of FOXF2-deficient iECs reveals widespread barrier and cellular transport deficits. **a**, Experimental outline for studying endothelial *FOXF2* deletion in human cells via proteomics. **b**, Volcano plot of log_2_(LFQ ratios) (KO versus WT) and −log_10_(*P* values) of all quantified proteins in iECs. Red and blue circles indicate proteins that were significantly upregulated and downregulated, respectively (*n* = 5 replicates per group; two-tailed Student’s *t*-test, *P* < 0.05). Proteins involved in cell adhesion and endocytosis are marked with their gene name. Dotted lines mark the FDR threshold (*P* < 0.05). **c**, Summary of the LC–MS/MS and LFQ results and enrichment analysis of biological processes of significantly dysregulated proteins in iECs based on the GO terms. **d**, Abundance of the top 60 significantly upregulated proteins related to the GO term endocytosis and downregulated proteins related to the GO term cell adhesion ordered alphabetically (*n* = 4–5 samples per group; Student’s *t*-test, *P* < 0.05). **e**, Increased uptake of FM1-43FX in FOXF2^KO^ iECs. Representative IF of iECs is shown with quantification of mean intensity of FM1-43FX (*n* = 10 replicates per group; normalized to WT; two-tailed Student’s *t*-test, *P* < 0.0001). Scale bar: 25 µm. **f**, Increased uptake of CTB in FOXF2^KO^ iECs. Representative IF of iECs is shown with quantification of mean intensity of CTB (*n* = 15 replicates per group; normalized to WT; comparison by two-tailed Student’s *t*-test, *P* < 0.001). Scale bar: 25 µm. **g**, TEER of FOXF2^KO^ iECs over 42 h quantified at 10 h (middle) and 30 h (right) (*n* = 3 replicates per group; normalized to WT; two-tailed Student’s *t*-test, *P* = 0.006, *P* = 0.01). **h**, Relative RNA abundance of *CLDN5*, *TJP1* and *OCLN* in iECs normalized to *EMC7* by qPCR (*n* = 5 replicates per sample; normalized to WT; two-tailed Student’s *t*-test, *P* < 0.001, *P* < 0.0001, *P* = 0.0034). **i**, Representative IF of 3D WT and FOXF2^KO^ cultures stained for TJP1 with quantification of junctions and diameter (Methods) (*n* = 3 replicates per group; normalized to WT; two-tailed Student’s *t*-test, *P* = 0.0056, *P* = 0.0035). Scale bar: 100 µm. **j**,**k**, Enrichment of FOXF2 (black) and H3K27ac (red) at *CAV1* (**j**) and *TJP1* (**k**) genes based on ChIP–seq analysis. The bar graphs show the mean ± s.d. count, total enriched proteins; cREs, *cis*-regulatory elements; dep., dependent; endo.-to-lyso., endosome-to-lysosome; LFQ, label-free quantification; *P*, significance of enriched terms. FE, fold enrichment; CTB, cholera toxin B subunit. Illustrations in **a** and **h** created with BioRender.com. Source data

To better understand the role of FOXF2 in BBB maintenance and explore the EC-specific molecular pathways mediated by FOXF2 we performed liquid chromatography–tandem mass spectrometry (LC-MS/MS)-based proteomics on human FOXF2^KO^ versus WT iECs (Fig. 4a). Proteomic analysis of ECs captured a total of 9,134 proteins identified with ≥2 unique peptides. Among them, 7,656 proteins were quantified in ≥3 samples and 2,839 were significantly altered. Out of these, 1,100 and 1,739 were upregulated and downregulated, respectively (Student’s *t*-test, *P* < 0.05) (Fig. 4b,c and Supplementary Table 5). In line with our results at the cellular level, GO enrichment analysis of significantly altered proteins revealed ‘vesicle-mediated transport’, ‘focal adhesion assembly’ and ‘regulation of vascular permeability’ among the most affected endothelial biological processes (Fig. 4c)—all highly regulated biological processes involved in the establishment and maintenance of BBB integrity.

Among the endocytosis proteins upregulated in FOXF2^KO^ human iECs, we found the main regulators of caveolar assembly, such as CAV1 and PACSIN2. Moreover, several members of clathrin-coated vesicles or cargo such as ACKR3, AP3M2, CLINT1, INPP5F and FCHSD2 were upregulated in our FOXF2-deficient iECs. In addition, expression of proteins known to be involved in intracellular protein transport, for example, in the Golgi vesicle transport system like EHD3 and RAB1A, were likewise upregulated, as were proteins involved in lipid transport (for example, GLUP1, LDLR and LRP1) and actin filament or cytoskeleton organization (for example, ARHGAP2, BIN3, CARMIL1, DPYSL2, EZR, GRB2 PDLIM7 and SH3P1) (Fig. 4d).

Focusing on cell adhesion, we found several proteins involved in the formation of tight junction complexes such as CDH5, CLDN5, ESAM, RAP2B, SYMPK and TJP1 to be downregulated. Proteins involved in cell adhesion mediated by integrin such as ITGA5, ITGA9, ITGB1BP1, MMRN1 and PLPP2 were also downregulated, as well as proteins involved in cell–matrix adhesion (for example, ADAM15, HPSE, JUP, TIMM10B and RAC1), cytoskeleton reorganization (for example, CORO2B, PIP5K1A, S1PR1 and TAOK2) and angiogenesis and vasculogenesis (for example, ENG2, EPHB4, NINJ1, NRP1, NRP2 and TIE2 (TEK)), suggesting an overall reorganization of cellular structure upon FOXF2 deletion (Fig. 4d).

**Extended Data Fig. 10:**
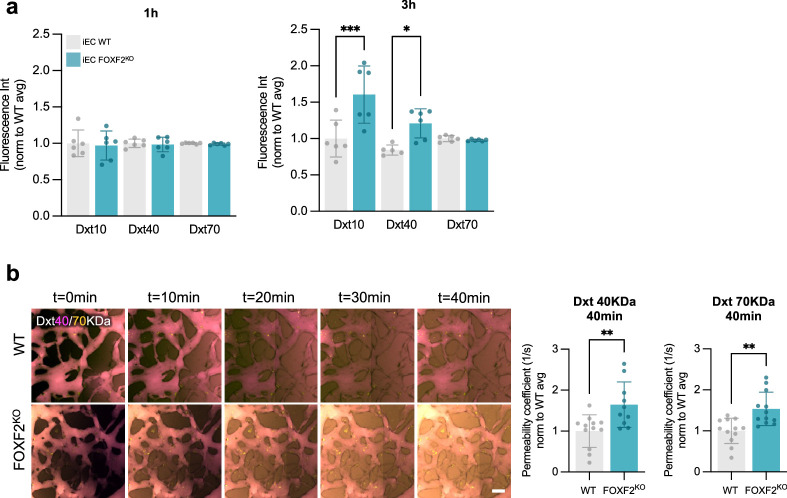
FOXF2 loss induces barrier deficits in 2D and 3D cultures. **a**. Dextran 10-KDa, 40-KDa and 70KDa leakage after 1 h and 3 h in WT and FOXF2^KO^ iECs measured by fluorescent intensity (normalized to WT) (n = 5-6 replicates/group; normalized to WT; one-way ANOVA corrected for multiple comparisons by Tukey’s method, P < 0,001, P=0.03). **b**. Dextran 40-KDa and 70-KDa leakage after 40 min in WT and FOXF2^KO^ 3D cultures together with representative live imaging pictures. Scale bar 50 µm (n = 10-12 replicates/group; normalized to WT; two-tailed T-test, P =0.0049, P=0.0015). Bar graphs show mean ± SD. Source data

To explore the functional consequences of FOXF2 deficiency on endothelial transport, we stained KO and WT human 3D cultures with FM1-43FX, a marker for endocytic vesicles^48^. FOXF2-deficient iECs exhibited a higher density of FM1-43FX-positive vesicles compared to WT iECs, suggesting an increased level of endocytic uptake on *FOXF2* deletion (Fig. 4e). To further understand whether the increased endocytosis is mediated by CAV1, we treated FOXF2-deficient 3D cultures with cholera toxin subunit B (CTB), which is taken up predominantly by CAV1-mediated endocytosis^49^. Indeed, FOXF2-deficient iECs showed an increased level of CTB uptake compared to WT (Fig. 4f). To explore whether the structural and molecular deficits in tight junctions also resulted in functional abnormalities in vitro, we measured the TEER of ECs seeded on transwells over time and found that FOXF2-deficient iECs showed a significant decrease in TEER compared to WT cells (Fig. 4g). These barrier deficits were accompanied by downregulation of tight junction transcripts, such as *TJP1*, *CLDN5* and *OCLN* in iECs (Fig. 4h and Extended Data Fig. 7h). Moreover, loss of FOXF2 also affected overall vessel complexity, because FOXF2^KO^ 3D-vessel-like tubes had fewer junctions and larger diameters (Fig. 4i). Last, we also found KO iECs more permeable to dextrans of different sizes in our 3D model. In comparison to transwell monoculture, this deficit was evident more rapidly and was more pronounced for larger dextrans, potentially indicating higher sensitivity and relevance of the 3D assay (Extended Data Fig. 10a,b). Together, these results—in line with our in vivo data^45^—show that endothelial-specific *Foxf2* deletion results in vessel abnormalities, BBB impairment and vascular leakage in both mouse and human systems.

To test the applicability of our human iECs to investigate a mechanistic link between FOXF2 transcription factor activity and the expression of FOXF2 target genes mediating transport and barrier function at the BBB, we turned to chromatin immunoprecipitation with sequencing (ChIP–seq) using an iPS cell line with doxycycline-inducible expression of *FOXF2* that we developed. Parsing a ChIP–seq dataset that we generated in a parallel study^45^, we found that FOXF2-binding peaks were enriched close to the transcriptional start site and along the coding region of *TJP1* and *CAV1*, respectively (Fig. 4j,k), suggesting that FOXF2 may directly regulate *TJP1* and *CAV1*, thus promoting EC stability to maintain a BBB phenotype in ECs. Overall, our analysis confirms the suitability of our human BBB model to recapitulate neurovascular disease phenotypes on FOXF2 loss and perform functional experiments to investigate disease mechanisms.

### LNP delivery of FOXF2 restores BBB deficits in vitro

**Fig. 5:**
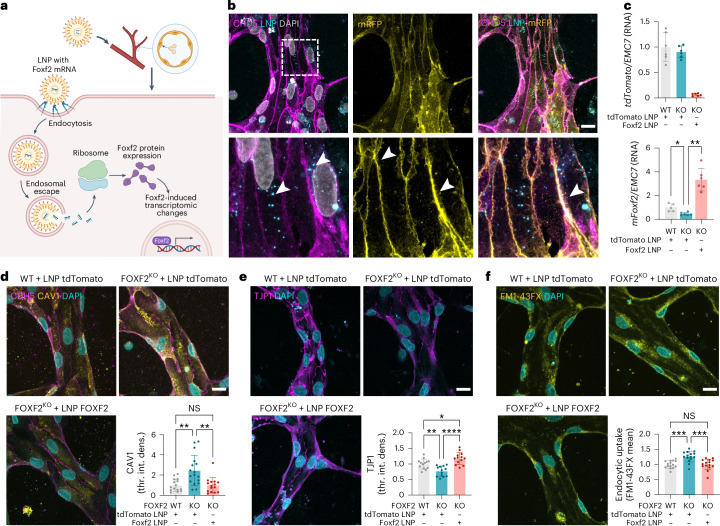
LNP delivery of Foxf2 restores caveolae and barrier function in the human 3D BBB model. **a**, Overview of LNP mode of action: uptake by ECs and *Foxf2* mRNA translation into protein. **b**, Representative IF of 3D cultures treated with mRFP LNPs. Scale bar: 25 µm. The arrows indicate fluorescein-labeled LNP particles (left) and mRFP expression at the membrane (middle) colocalizing with CDH5 (right). **c**, Relative RNA abundance of *tdTomato* Ctrl LNP (top) and *Foxf2* (bottom) in iECs after LNP treatment, normalized to *EMC7* (*tdTomato*/control versus *Foxf2* LNP treatment) measured by qPCR (*n*  = 5–6 replicates per sample; normalized to WT-tdTomato treatment; two-tailed Student’s *t*-test, *P*  = 0.03, *P* = 0.001). **d**,**e**, Representative IF of FOXF2-deficient 3D cultures treated with LNPs and stained for CAV1 (**d**) or TJP1 (**e**), with quantification of CAV1 (int. dens.) or TJP1 (int. dens.) (*n* = 13–17 replicates per group; normalized to WT-tdTomato treatment; one-way ANOVA corrected for multiple comparisons by Tukey’s method; **d**, *P* > 0.99, *P* = 0.001; **e**, *P* = 0.029, *P* = 0,0043, *P* < 0.0001). Scale bar: 25 µm. **f**, Endocytic uptake of FM1-43FX in iECs after LNP treatment (tdTomato or control versus Foxf2, one-way ANOVA corrected for multiple comparisons by Tukey’s method, *P* > 0.99, *P* < 0.001). Representative IF of iECs was shown in 3D culture with quantification of mean intensity of FM1-43FX (relative to WT) (*n* = 15 replicates per group). Scale bar: 25 µm. The bar graphs show mean ± s.d. Illustrations in **a** created with BioRender.com. Source data

To assess the applicability of our human BBB model for testing therapeutic modulations, we treated FOXF2-deficient cultures with LNPs containing *Foxf2* mRNA. We reasoned that, after LNP uptake via endocytosis and endosomal escape, therapeutically delivered mRNA would be translated into protein and restore FOXF2 expression (Fig. 5a). We first tested and optimized delivery of fluorescein-labeled LNPs containing membrane-RFP (mRFP) mRNA through the lumen of the vessel-like tubes, which led to broad mRFP expression in most iECs and colocalization with CDH5, an endothelial adhesion marker (Fig. 5b), indicating high efficiency of delivery. Notably, only iECs, but not other cell types, expressed mRFP, indicating that the LNPs do not pass the barrier formed by the cells, consistent with in vivo conditions^50^. On treatment with control (*tdTomato*) or *Foxf2* LNPs, WT and FOXF2-deficient iECs showed increased *tdTomato* or *Foxf2* mRNA levels, respectively, demonstrating successful delivery of these transcripts into iECs (Fig. 5c). Strikingly, *Foxf2*-LNP-treated, FOXF2-deficient 3D cultures downregulated CAV1 (Fig. 5d) and upregulated TJP1 (Fig. 5e) protein to levels similar to the WT condition. Moreover, Foxf2 overexpression via LNPs reduced the endocytic uptake of FM1-43FX in KO cultures, thus rescuing both molecular and functional phenotypes caused by FOXF2 deficiency (Fig. 5f). Together, the LNP data suggest that our 3D BBB model may be a useful tool to test interventions of therapeutic agents modulating the endothelium in vitro.

## Discussion

The ability to study BBB physiology and dysfunction critically depends on model systems suitable for functional experiments and the importance of developing and applying in vitro models derived from human cells is increasingly recognized^6^. Here we developed a fully human iPS-cell-derived in vitro BBB model composed of iPS-cell-derived ECs, mural cells and astrocytes and demonstrated its suitability for studying neurovascular disorders in an isogenic context. Although several iPS cell differentiation protocols have been developed to generate BBB cell types, further improvements in standardization and validation are needed to more precisely define cellular identities^51^. This will help to avoid the use of misidentified populations for BBB modeling, such as epithelial cells^11,30^, which have mistakenly been used in a remarkable number of studies (Supplementary Table 3). Multi-level characterization of our iPS-cell-derived iECs, iPCs, iSMCs and iASs revealed distinct, well-defined populations of BBB cell types. The iEC expression profiles more closely resembled brain endothelium than other endothelia or epithelia, and upregulation of cell-specific processes and performance in functional assays assessing transporters and TEER confirmed brain endothelial identity. Our data also indicate that primary ECs may not accurately reflect in vivo conditions, because they showed inferior performance in expression and functional studies compared to hCMECs and HUVECs, which we used in addition for a more faithful cell-type validation. The iASs exhibited typical astroglial identity at morphological and gene expression levels. A critical factor for the generation of nonreactive iASs supporting BBB formation in vitro was their differentiation under serum-free conditions^26^. The iPCs and iSMCs also expressed typical mural cell markers and some separation based on morphology and expression profiles, but overall remained quite similar to each other. Defining mural cell identities and distinguishing between pericytes and SMCs have been challenging for the entire field due to their heterogeneous distribution along the vascular tree^52^ and the lack of specific markers defining different mural cell types^1,53^. Moreover, identity of mural cells may be determined or influenced by interactions with ECs, ASs and other neighbors in their individual niches in vivo, leading to de-differentiation and loss of specific identity markers when extracted from the brain and cultured. For the same reason, mural cells coming out of iPS cell differentiation protocols may lack some key factors for final maturation and separation into distinct mural cell types, limiting the potential of in vitro systems to fully model mural cell biology.

Furthermore, the developmental source of mural cells also remains insufficiently understood. Although early findings suggested mesodermal origin^54^, subsequent studies using lineage-tracing and quail-chick chimeras revealed that mural cells in the forebrain descend from both mesoderm and ectodermal neural crest^53,55,56^. Moreover, some hematopoietic lineages can give rise to brain pericytes^57^, further illustrating heterogeneity in the cellular origin. Our data show that both induced and primary SMCs and pericytes cluster closely together in PCA and express cell-type markers at comparable levels. Some PC markers were higher in pericytes versus SMCs and vice versa, but none of the markers was exclusively expressed in one of the cell types. Comparison with primary cells and published RNA-seq datasets revealed that this phenomenon is not limited to induced cells, but also visible ex vivo and in vivo. Overall, even though our analysis suggests successful differentiation of mural cells expressing typical markers and separation of iPCs and iSMCs into distinct populations^53,58^, more work is needed to define the identities of mural cell types and optimize their differentiations from iPS cells into more in vivo-like populations. Future studies should also investigate the influence of the niche and optimize in vitro systems to better emulate such niches. Finally, to facilitate human BBB cell characterization and enable identification of further cell-specific markers in future studies, we provided all proteomics profiles in an interactive database for NeuroVascular cell Proteins (available at https://dbNeuroVasP.isd-muc.de) as a resource for the field.

Applying our well-characterized BBB cell types, we next established a fully human in vitro 3D BBB model, in which iECs self-organized into vessel-like structures with correct topology, cell–cell interactions, secreted extracellular matrix proteins and perfused lumina in a microfluidic chip. Most earlier models either lacked certain cell types^59^ or combined iPS-cell-derived with primary cells^13,15^, which introduces complications due to nonisogenic backgrounds and limits reproducibility. We further explored the cell-autonomous effect of FOXF2 loss on BBB cell types using FOXF2-deficient human iECs differentiated from genome-edited iPS cells. BECs present with a low rate of transcytosis and high expression of tight junction proteins^4^ and both were affected in our FOXF2-deficient human in vitro system: indeed, our model phenocopied key BBB phenotypes found in vivo, including elevated caveolae formation, downregulation of TJP1 and malformation of tight junctions on loss of FOXF2. Moreover, the model allowed performing functional assays difficult to assess in vivo, such as determining endocytic uptake and identifying the transport systems involved. In these assays, FOXF2 loss induced increased endocytic uptake via caveolae and, in addition, decreased TEER, suggesting an involvement of endothelial FOXF2 in regulating vesicle-mediated transport and cell junction integrity.

Strikingly, loss of FOXF2 resulted in formation of a less complex vascular network, as well as leakage of vessels in 3D, which phenocopies our findings in vivo^45^ and thus demonstrates the advantages of the 3D model over available 2D models. To dive deeper into the mechanisms mediating loss of FOXF2, we performed proteomics of FOXF2^KO^ iECs, which revealed vesicle-mediated transport, focal adhesion assembly and regulation of vascular permeability among the most affected endothelial biological processes in human FOXF2-deficient iECs, fully in line with the cell biological deficits that we found. Consistent with other genetic models, where increased caveolin-mediated transport has been linked to BBB leakage^60^, we found several dysregulated proteins involved in caveolae formation, such as CAV1. Moreover, several molecular constituents of clathrin-coated vesicles and intracellular protein transport were upregulated. Together, these changes may contribute to the increased endocytic uptake in FOXF2^KO^ cultures. In addition, several proteins involved in cell adhesion were downregulated, including those involved in formation of tight junction complexes such as TJP1, OCLN and CLDN5. These results are in line with previously published data in hypoxia-induced brain injury, in which a leaky BBB developed with decreased expression and reorganization of these main tight junction proteins^61^. Furthermore, several neurological disorders presenting with BBB breakdown have been associated with reduced TJP1 levels^62^. Finally, proteomics and ChIP–seq results suggested a direct effect of FOXF2 on TJP1 and CAV1 expression by binding to the locus.

As a last step, we developed a rescue paradigm for FOXF2 loss using LNPs. Perfusing FOXF2^KO^ cultures with LNPs containing *Foxf2* mRNA restored the levels of CAV1 and TJP1 and reduced endocytic uptake of FOXF2^KO^ cultures and WT cultures, further supporting a direct involvement of FOXF2 in endocytosis regulation. These experiments not only demonstrate the applicability of the BBB model for testing translational and therapeutic delivery approaches to ECs, but also highlight its advantages over simpler 2D culture systems like transwells, by delivering LNPs directly into 3D vessels under flow. Furthermore, the system can be scaled up for screening using a pipetting robot, as demonstrated by the supplier. LNPs can also be decorated with antibodies or other moieties for cell-specific delivery across the BBB^50^ and the expression of pharmacologically targeted surface receptors like TFRC^36–38^ may enable testing additional strategies for therapeutic delivery into or across the BBB.

As a limitation, our 3D BBB model currently does not recapitulate constant physiological flow to induce shear stress in the vessel-like tubes. Shear stress influences endothelial BBB phenotypes by reducing apoptotic pathways^63^, increases membrane transporters and tight junctions such as TJP1 and OCLN and further limits permeability^64^. Unlike in systems where ECs grow on predefined surfaces, our model relies on angiogenesis in the soft matrix of the microfluidic chip, generating more physiological vessel-like structures amenable to shear stress. However, this process may introduce variability in the vessel dimensions, making comparisons between different samples challenging. Moreover, the microfluidic chips used do not support live measurement of certain physiological parameters, such as TEER, due to the lack of sensors, which limits the investigation of BBB permeability. The observed TEER values were higher than in previous studies but did not yet reach the values reported in vivo. Furthermore, the small size of the microfluidic devices limits proteomics and transcriptomics studies. Future technical improvements will mitigate these limitations^28,29^. As our study focused on investigating FOXF2 deficiency in ECs only, future studies should also explore its loss in the second BBB cell type expressing it, pericytes. This would enable the identification of potential noncell-autonomous effects of pericytes on ECs upon FOXF2 loss. Finally, incorporating different genetic backgrounds and additional cell types into the iPS cell BBB model, such as microglia, neurons and monocytes, will be crucial for comprehensively studying biological variability, as well as the complexity of the BBB, including cellular crosstalk and inflammatory responses.

## Methods

### Culture of iPS cells

All iPS cell experiments were performed according to all relevant local guidelines and regulations. All work was done with the commercially available female iPS cell line (Thermo Fisher Scientific, cat. no. A18945, hPS cell reg. name TMOi001-A, RRID:CVCL_RM92). The iPS cells were maintained on vitronectin-coated culture plates (Thermo Fisher Scientific, cat. no. A14700, diluted 1:100 in phosphate-buffered saline (PBS), for 1 h at room temperature (RT)) and Essential 8 Flex Medium (E8F; Thermo Fisher Scientific, cat. no. A2858501) at 37 °C with 5% CO_2_ until they reached 80% confluency. Cells were routinely passaged using PBS with 500 nM EDTA (Thermo Fisher Scientific, cat. no. 15575020) for 5 min and plated again in E8F.

### CRISPR–Cas9 genome editing

CRISPR–Cas9 editing was performed as described previously^65,66^, with modifications for ribonucleoprotein (RNP)-based DNA cleavage^67^. Briefly, cells were dissociated in preparation for electroporation using Accutase (Thermo Fisher Scientific, cat. no. A1110501), plated in Geltrex-coated (Thermo Fisher Scientific, cat. no. A1413302) culture plates in StemFlex (SF; Thermo Fisher Scientific, cat. no. A3349401) with 10 µM ROCK inhibitor (RI; Selleckchem, cat. no. S1049) at a density of 150,000 cells cm^−2^ and cultured for 2 d. To prepare the RNP complex, 60 pmol of small guide RNA targeting *FOXF2* (ttcttccgcggcgcctaccaggg, ordered from Synthego) was mixed with 30 pmol of high-fidelity Cas9 mutant (IDT, cat. no. 1081060) and incubated at RT for 10–20 min. After Accutase dissociation, 200,000 cells were resuspended in 20 µl of P3 Primary Cell Nucleofector Solution (Lonza, cat. no. V4XP-3032) and gently mixed with the incubated RNP complex. The mixture was transferred into one well of a nucleocuvette strip (Lonza, cat. no. V4XP-3032) and cells were electroporated in a 4D Nucleofactor X Unit (Lonza, cat. no. AAF-1002X) using program CA137. After electroporation, cells were plated in one 12-well Geltrex-coated culture plate with SF supplemented with 1× RevitaCell (Thermo Fisher Scientific, cat. no. A2644501) and grown for 2–4 d. Cells were then plated at low density and single-cell clone colonies were picked, analyzed by restriction fragment length polymorphism using the enzyme BstNI (New England Biolabs (NEB), cat. no. R0168S) and Sanger sequencing as previously described^66^. The knockout was confirmed at the RNA level using real-time qPCR analysis. For quality controls, pluripotency was confirmed via IF using OCT4, NANOG, SSEA4 and TRA16 and chromosomal integrity was validated by molecular karyotyping (LIFE & BRAIN GmbH). Off-target analysis was performed by Sanger sequencing on the top five most likely loci based on MIT and cutting frequency determination (CFD) scores predicted by the CRISPOR (http://crispor.tefor.net/) guide RNA design tool^68^. On-target effects, such as loss of heterozygosity, were also analyzed using nearby SNP sequencing as previously described^43^.

### Somatic cell differentiation of iPS cells

#### Endothelial cells (iECs)

The iPS cells were seeded on to Geltrex-coated (Thermo Fisher Scientific, cat. no. A1413201, 1:150 in Dulbecco’s modified Eagle’s medium (DMEM)/F12, Thermo Fisher Scientific, cat. no. 11320033, for 1 h at 37 °C) culture plates at a density of 200,000 cells cm^−2^ in SF medium with 10 µM RI for 24 h. Medium was replenished every 24 h for the next 5 d. On days 1–2, cells were fed with Mesoderm Induction Medium (STEMCELL, cat. no. 05220) followed by APEL2 medium (STEMCELL, cat. no. 05270) with 200 ng ml^−1^ of VEGF (Peprotech, cat. no. 100-20) and 2 µM forskolin (Peprotech, cat. no. 6652995) on days 3–4. On day 5, ECs were selected by CDH5 MACS. After Accutase dissociation, cells were incubated with CDH5 Microbeads (Miltenyi Biotec, cat. no. 130-097-867) for 15 min at 4 °C. The CDH5^+^ fraction was obtained via MACS following the manufacturer’s instructions and plated on to Collagen IV-coated (Sigma-Aldrich, cat. no. C5533-5MG, 1:40 in PBS, for 1 h at 37 °C) culture plates at a density of 200,000 cells cm^−2^ in EC medium (ECM; PromoCell, cat. no. C-22011) supplemented with 50 ng ml^−1^ of VEGF. iECs were grown until they reached 80–90% confluency, but avoiding 100%, and passaged with trypsin-EDTA (Thermo Fisher Scientific, cat. no. 25200056) in a ratio of 1:2–1:6 for up to 5 passages.

#### Smooth muscle cells (iSMCs)

The iPS cells were seeded at a density of 200,000 cells cm^−2^ on to Geltrex-coated culture plates with SF medium and 10 µM RI. Differentiation was started 24 h after seeding by switching medium to Mesoderm Induction Medium. Medium was replenished every 24 h for 3 d consecutively. On day 4, medium was switched to APEL2 medium supplemented with 50 ng ml^−1^ of VEGF and 25 ng ml^−1^ of BMP4 (Peprotech, cat. no. AF 120-05ET) and replenished every second day. On day 8, cells were split in a ratio 1:4–1:6 on to Collagen IV-coated culture plates with SMC medium (Promocell, cat. no. C-22062) with 10 ng ml^−1^ of platelet-derived growth factor-BB (PDGFBB; Peprotech, cat. no. 100-14B) and 2 ng ml^−1^ of transforming growth factor β1 (TGFB1; Peprotech, cat. no. AF-100-21C). Cells were passaged on confluency using trypsin-EDTA in a ratio of 1:2–1:8 for up to 5 passages.

#### Pericytes (iPCs)

The iPS-cell-derived pericytes were generated as previously described^25^ with some modifications. Days 1–5 of the differentiation are identical to the EC protocol above. After the CDH5 cell selection via MACS, the negative fraction, CDH5^−^, was plated on to gelatin-coated (Merck Millipore, cat. no. ES-006-B, for 1 h at 37 °C) culture plates with ECM at a density of 200,000 cells cm^−2^. At 90% confluency, cells were split on to gelatin-coated culture plates with DMEM/GlutaMAX (Thermo Fisher Scientific, cat. no. 10566016) supplemented with 10% fetal bovine serum (FBS; Biowest, cat. no. S1860), 2 ng ml^−1^ of TGFB3 (Peprotech, cat. no. 100-36E) and 4 ng ml^−1^ of PDGFBB in a ratio of 1:1-1:2 using TrypLE (Thermo Fisher Scientific, cat. no. 12604013). The medium was changed after 3 d to DMEM–10% FBS without growth factors.

#### Astrocytes (iASs)

The iPS-cell-derived astrocytes were generated as previously described^26^ with some modifications. The iPS cells were first differentiated into neural precursor cells (NPCs) as described previously^69^, followed by glia induction and maturation. To generate NPCs, iPS cells were split into single cells using Accutase and seeded on to poly(L-ornithine) (P)-laminin-coated (pOL) plates at a density of 0.3 million cells cm^−2^ in neural induction medium (NI) with 10 μM RI. NI is composed of neural maintenance medium (NM) supplemented with 10 mM SB-431543 (Selleckchem, cat. no. S1067) and 2.5 mM LDN-193189 (Selleckchem, cat. no. S2617). NM is composed of 0.5× neurobasal medium (Thermo Fisher Scientific, cat. no. 211003-049), 0.5× DMEM/F12 (Thermo Fisher Scientific, cat. no. 11320-074) supplemented with 100 U ml^−1^ of penicillin–streptomycin (Thermo Fisher Scientific, cat. no. 12140-122), 0.5× B27 supplement with vitamin A (Thermo Fisher Scientific, cat. no. 17504044), 2 mM GlutaMAX (Thermo Fisher Scientific, cat. no. 35050), 1× NEAA (Thermo Fisher Scientific, cat. no. 11140-050), 0.5× N-2 supplement (Thermo Fisher Scientific, cat. no. 17502048), 1.5 μg ml^−1^ of insulin (Sigma-Aldrich, cat. no. 10515) and 0.05 mM 2-mercaptoethanol (Thermo Fisher Scientific, cat. no. 21985-023). NI medium was replenished every 24 h until DIV7. On DIV7, cells were split on to pOL-coated plates. The pOL-coated plates were generated by coating first with pOL (Sigma-Aldrich, cat. no. P4957, diluted 1:100 in ddH_2_O) for 4 h followed by laminin (Thermo Fisher Scientific, cat. no. 23017015, diluted 1:100 in PBS) overnight at RT. Before cell-dissociated, laminin-coated solution was aspirated, plates were dried in a laminar flow hood for at least 30 min. Cells were dissociated with Accutase and resuspended with NI + RI at a density of 30 million cells ml^−1^. From the cell suspension, 250 µl was slowly added to the pOL-coated plate to form a spot. Spots were incubated for 1 h at RT to let the cells attach and then fed with NI + RI. Cells were fed regularly with NI until DIV10, when the medium was changed to NM supplemented with 20 ng ml^−1^ of bFGF (STEMCELL Technologies, cat. no. 78003.2) until DIV12. Cells were further cultured with NM until the first neural rosettes formed, around DIV14–15. On DIV15, medium was changed to glial precursor expansion medium (GEM), composed of DMEM/F12 supplemented with 1× GlutaMAX (Thermo Fisher Scientific, cat. no. 35050061), 1× N-2 supplement (Thermo Fisher Scientific, cat. no. 17502048), 1× B27 supplement (Thermo Fisher Scientific, cat. no. 12587010), 10 ng ml^−1^ of FGF-2 and 10 g ml^−1^ of EGF (Peprotech, cat. no. 100-15-100). The medium was exchanged every day until DIV17.

Rosettes were split using Neural Rosette Selection agent (NRSR, STEMCELL Technologies, cat. no. 5831). Cells were incubated with NRSR for 60–90 min at 37 °C and rosettes from the center of the spot were manually isolated. After brief centrifugation, cells were resuspended in GEM and plated on to pOL-coated plates. When cells reached confluency, they were further split using Accutase at a density of 0.1 million cells cm^−^^2^ on to gelatin-coated plates with GEM. Medium was replenished every day and wells were further split using Accutase and GEM when confluent. On DIV20, medium was changed to astrocyte induction medium (AIM), composed of DMEM/F12 supplemented with 1× penicillin–streptomycin, 1× GlutaMAX, 1× N-supplement, 1× B27 supplement without vitamin A, 10 ng ml^−1^ of EGF and 10 ng ml^−1^ of LIF (Peprotech, cat. no. 300-05-26). When cells were confluent, they were subsequently split using Accutase and plated at a density of 0.1 million cells cm^−^^2^ on to GT-coated plates. On DIV34, medium was changed to astrocyte medium (AM) with 50 µg ml^−1^ of CNTF (Peprotech, cat. no. 450-13-20). AM contains DMEM/F12 supplemented with 1× penicillin–streptomycin, 1× B27 supplement, 1× GlutaMAX and 50 µg ml^−1^ of CNTF. Cells were fed with AM + CNTF for 28 d more, then the medium on DIV62 was changed to AM. The purity of the astrocytes was carefully checked before use.

### Primary EC, PC, SMC and AS culture

Human brain microvascular ECs (HBMECs, cat. no. 1000; also called primary endothelial cells, pECs), human brain vascular pericytes (HBVPs, cat. no. 1200; also called primary pericytes, pPCs), human brain vascular smooth muscle cells (HBVSMCs, cat. no. 1100, also called primary smooth muscle cells, pSMCs) and human astrocytes (HAs, cat. no. 1800; also called primary astrocytes, pASs) and human umbilical vein endothelial cells (HUVECs, cat. no. 8000) were purchased from ScienceCell. Human cerebral microvascular endothelial cells or BBB line (hCMECs or D3) was purchased from Merck (cat. no. SCC066). HBMECs or pECs and HUVECs were cultured as our iECs, in ECM (supplemented with 50 ng ml^−1^ of VEGF, whereas hCMECs were cultured only in ECM without VEGF addition). All ECs were seeded on to Collagen IV-coated plates. HBVPs or pECs were cultured as our iPCs, in DMEM/GlutaMAX supplemented with 10% FBS on to gelatin-coated plates. HBVSMCs or pSMCs were cultured as our iSMCs in SMC medium and on to Collagen IV-coated plates. HAs or pASs were also cultured as our iASs using AM medium and on to Geltrex-coated plates.

### TEER assay

TEER was measured using either the Nanoanalytics CellZscope system or the Merk Milicell ERS 3.0 Digital Voltohmmeter according to the manufacturer’s instructions. For co-culture, mural cells (iPCs or iSMCs) and astrocytes were split on to the basal side of Geltrex-coated transwells (Corning, cat. no. 353095) on day 1 and incubated at 37 °C and 5% CO_2_ overnight. On day 2, ECs were split on to the apical side of Collagen IV-coated transwells and incubated for 2 d at 37 °C and 5% CO_2_. On day 4, cultures were mounted in the CellZscope system, placed at 37 °C and 5% CO_2_, or measured with the Milicell system at 35–37 °C. For monoculture, ECs were split directly on to the apical side of the transwells on day 1 and measurements were started on day 3. Cells were cultured with VASC medium (composed of two-thirds ECM without supplement and one-third AM).

### ABC transporter assay

ABC transporter function was assessed as previously reported^39,40^. The functionality of the P-GP (*ABCB1*), MRP1 (*ABCC1*) and BCRP1 (*ABCG2*) transporters was measured by fluorescent transport of substrates across an EC monolayer in a transwell insert. We used the following substrates specific for each transporter: 2 µM rhodamine-123 (Invitrogen, cat. no. R302), 10 µM doxorubicin (Sigma-Aldrich, cat. no. D1515-10MG) and 10 mM 2′,7′-dichlorofluorescin diacetate (DCFDA; Thermo Fisher Scientific, cat. no. D399). We also used specific inhibitors: 50 µM verapamil (Sigma-Aldrich, cat. no. V4629-1G), 1 µM Ko 143 (Sigma-Aldrich, cat. no. K2144-1MG) and 10 mM MK571 (Sigma-Aldrich, cat. no. M7571-5MG). Cells were pre-treated for 1 h with inhibitors and then incubated with substrates or substrates and inhibitors for 4 h using pheno-red-free ECM (PromoCell, cat. no. C-22216). Medium in the bottom compartment was collected and fluorescent intensity was measured on a plate reader (Thermo Fisher Scientific, Fluoroskan Ascent; 485-nm excitation and 538-nm emission for rhodamine, 485-nm excitation and 510-nm emission for doxorubicin and 530-nm excitation and 590-nm emission for DCFDA).

### Dextran assay in two dimensions

Leakage of the EC layer was assessed using a transwell system. Cells, upon 100% confluency, were incubated with 0.25 mg ml^−1^ for 1–3 h of different dextran sizes: Dxt-10 kDa (Invitrogen, cat. no. D1820), Dxt-40 kDa (Invitrogen, cat. no. D1845) and Dxt-70 kDa (Invitrogen, cat. no. D1864). Medium at the bottom compartment was collected and fluorescent intensity was measured on a plate reader (485-nm excitation and 538-nm excitation for FITCs and 530-nm excitation and 590-nm emission for Texas Red).

### Generation of microfluidic, 3D in vitro BBB model

The iPS-cell-derived ECs, pericytes, SMCs and ASs were used between passages 1 and 4. The 3D BBB model was generated using microfluidic chips (AIM Biotech, cat. no. DAX-1) following principles described previously^13^. Cells were detached following their respective protocols and resuspended in ECM containing 9 U ml^−1^ of thrombin (Sigma-Aldrich, cat. no. F8630). The following cell ratios were used: 1.2 million per ml of ECs, 0.5 million per ml of ASs and 0.1 million per ml of pericytes or SMCs. Cell suspension was mixed in a 1:1 ratio with a 6 mg ml^−1^ of fibrinogen solution in PBS (Sigma-Aldrich, cat. no. F8630) and immediately injected into the gel-filling ports (defined as day 1). Microfluidic devices were placed in a humidified chamber and polymerized at RT for 15 min. After gel polymerization, cultures were feed with VASC medium composed of two-thirds ECM without supplement (PromoCell, cat. no. C-22011) and one-third AM supplemented with 50 ng ml^−1^ of VEGF (Peprotech, cat. no. 100-20) for the first 4 d, then VEGF was removed. On day 2, the medium channels were coated with an EC monolayer by seeding the cells at a concentration of 1.5 million per ml. Gravity-driven flow was induced by feeding 70 µl on to the right media port and 50 µl on to the left port. Cultures were fed daily, incubated at 37 °C and 5% CO_2_ and used for experiments on day 5. Monocultures had a success rate of around 95%, co-cultures including two cell types (iECs + iPCs, iECs + iSMCs or iECs + iASs) had a success rate of around 80% and co-cultures including three cell types (iECs + iASs + iPCs or iECs + iASs + iSMCs) had a success rate of around 70%, with cell quality being the most important factor for 3D culture generation.

### Dextran assay in 3D model

To assess EC junction formation and 3D BBB model permeability, a mixture of 10-kDa dextran (Invitrogen, cat. no. D1976) and 40-kDa dextran (Invitrogen, cat. no. D1845) as fluorescent tracers was diluted in ECM and filled into the media ports under a confocal microscope as previously described^13,29^. In brief, after 5 d of culture, microfluidic devices were placed into an environmental conditioning chamber set to 37 °C and 5% CO_2_ mounted on a confocal microscope. Culture medium was carefully aspirated only from one media port, imaging was started and dextran solution was injected into the media port. Confocal images were acquired every 3 min 27× to create an entire 3D maximum projection of the microfluidic device for the TJ validations and every 10 min 5× for comparing WT versus FOXF2^KO^ cultures. Only areas with functional perfusion from time *t* = 0 were selected; regions showing leakage from *t* = 0, for example, due to insufficient seeding of side channels or technical deficits of the chip, were excluded.

### Calcium imaging in the 3D model

Cells were loaded with Fluo-4 (AAT Bioquest, cat. no. 271-20551) for 30 min at 37 °C and subsequently placed on an inverted microscope (Axio Observer Z1, Zeiss). Cells were excited at 470 ± 20 nm with 200 Hz and emission was collected with a bandpass filter at 525 ± 25 nm (ET470/40x; T495lpxr; ET525/50 m, Chroma). The intensities of the excitation light and exposure time were minimized to avoid phototoxicity while maintaining sufficient signal-to-noise ratio. Cells were imaged every 5 s while being superfused with 5% CO_2_ and 95% air in ECM. To better quantify the temporal existence (duration) of individual Ca^2+^ events, we reconstructed them as 3D spatiotemporal objects. This was achieved by grouping events in multiple frames based on their degree of spatial overlap. We extracted basic parameters of duration (s), maximum footprint area (µm^2^) and average and maximum intensity (*z*-score) from these objects.

### FM1-43FX and CTB treatment in the 3D model

Cells were incubated with 5 μg ml^−1^ of FM1-43FX (Thermo Fisher Scientific, cat. no. F35355) or CTB (Thermo Fisher Scientific, cat. no. C22841) diluted in ddH_2_O and sterile PBS corresponding to 30 min at 37 °C. After incubation cultures were washed twice with PBS and fixed with 4% paraformaldehyde (PFA) for further analysis.

### TNF treatment in two and three dimensions

Cells were incubated with 100 ng ml^−1^ of TNF (Peprotech, cat. no. 300-01A-50UG) at 37 °C for 6 h for immunohistochemistry analysis or 24 h for TEER analysis. After incubation, cultures were washed with PBS and fixed with 4% PFA or the medium was changed without TNF supplementation for TEER measurements.

### LNP generation and treatment

The LNPs (LNP-mRNA) were prepared by ISAR Bioscience GmbH. A plasmid expressing mouse *Foxf2* (Origene, cat. no. MR226011) was used for the generation of *Foxf2* LNPs, to allow distinction between exogenous and endogenous expression in qPCR assays. Note that, due to high conservation of the locus, the mouse *Foxf2* qPCR primer still has a minor affinity for human *FOXF2*.

#### Generation and purification of mRNA

The mRNA used for the formulation of the LNP was prepared in house using in vitro transcription of a template DNA. For this, the HiScribe T7 mRNA Kit with CleanCap Reagent AG from NEB—an optimized RNA synthesis formulation and trinucleotide cap analog technology for co-transcriptional capping of mRNAs—was used. After in vitro transcription, an additional enzymatic A-tailing was performed to ensure the stability of the mRNA. The linearized plasmid (tdTomato linearized with Xba1) and all kit components apart from the enzymes were thawed to RT. For one reaction, 1 µg of template DNA, each 2 µl of reaction buffer (10×), CleanCap Reagent AG (40 mM) and NTPs (50 mM) were mixed in a PCR tube. Here UTP was substituted with 1-*N*-methylpseudouridine (CrystalChem). Subsequently, 2 µl of T7 RNA polymerase was added and the reaction incubated at 37 °C for 2 h in a thermocycler (AnalytikJena, cat. no. 846-x-070-311). Afterwards 2 µl of DNase1 was added to the reaction and incubated for 15 min at 37 °C. For the A-tailing, 63 µl of nuclease-free water, 10 µl of 10× PolyA Reaction Buffer (NEB, cat. no. B0276S) and 5 µl of Poly(A) Polymerase (NEB, cat. no. ML0276L) was added to the 20-µl reaction and incubated for 30 min at 37 °C. Subsequently, the mRNA was cleaned using the Monarch RNA Cleanup Kit (NEB, cat. no. T2050L). The synthesized mRNA was purified using the Monarch RNA Cleanup Kit, 500 µg. To 100 µl of mRNA reaction, 200 µl of binding buffer and 300 µl of absolute ethanol (VWR, cat. no. 20821.330) were added and mixed by pipetting up and down. The sample was then loaded on to the column which was inserted in the collection tube and centrifuged at 16,000*g* for 1 min. The flow-through was discarded and the column washed twice with 500 µl of RNA Cleanup Wash Buffer (16,000*g* for 1 min). The column was transferred into an RNase-free 1.5-ml microfuge tube and the cleaned mRNA eluted with 50 µl of nuclease-free water after an incubation time of 5 min at RT by centrifuging the tube for 1 min at 16,000*g*. The purified mRNA was stored at −80 °C until further use.

#### LNP formulation

The LNPs were prepared using NanoAssemblr (Precision Nanosystems) microfluidic mixing technology under time invariant conditions: 1 ml of an aqueous solution containing mRNA at a concentration of 174 µg ml^−1^ in aqueous 70 mM acetate buffer, pH 4.0, was mixed with 0.4 ml of aqueous ethanolic lipid solution containing 12.5 mM lipids to form the nanoparticles. The flow rate ratio between the aqueous solution and the aqueous ethanolic lipid solution was 3:1 and the total flow rate was 12 ml min^−1^. The aqueous ethanolic lipid solution was prepared by dissolving 6-((2-hexyldecanoyl)oxy)-*N*-(6-((2-hexyldecanoyl)oxy)hexyl)-*N*-(4-hydroxybutyl)hexan-1-aminium (ALC-0315, Avanti, cat. no. 890900O), 1,2-dioleoyl-*sn*-glycero-3-phosphoethanolamine (DOPE, Avanti Polar Lipids cat. no. 850725P) or DOPE-fluorescein (MedChemExpress, cat. no. HY-D1556), cholesterol (Merck, cat. no. C8667) and methoxypolyethyleneglycoloxy(2000)-*N*,*N*-ditetradecylacetamide (ALC-0159; Avanti Polar Lipids, cat. no. 8801585 P) in a molar ratio of 50:10:38.5:1.5 in ethanol. This may be done by preparing separate 12.5 mM solutions of each lipid in the ethanol and mixing the solutions in the ratio given above to give the aqueous ethanolic lipid solution.

Then, 1.0 ml of the obtained LNP-mRNA product was immediately diluted with 40 ml of PBS (1×) and concentrated to 1.0 ml at 2,000g for 30 min at 20 °C using Amicon Ultra-15 centrifugal filtration tubes. Finally, the LNP sample was sterilized with a 0.2-µm syringe filter and stored at 4 °C. The size distribution of particles and polydispersity index were measured using Zetasizer Ultra (Malvern Panalytical Ltd). The LNP formulations used are listed in Supplementary Table 6.

#### LNP treatment in vitro

LNPs were diluted in ECM (1:100 (3D cultures) and 1:1,000 (2D monolayers)) and added to the cells. LNPs were incubated for 24 h. After treatment, cultures were washed twice with PBS and fixed with 4% PFA for further analysis.

### Analysis of the 3D-vessel network

#### Whole-network diameter and branching analysis

Image analysis was done in Fiji/ImageJ (1.52p and 1.54f). Intercellular space was manually removed by an imaging expert. Then the cells were binarized and subjected to a four-step iterative morphological erosion and dilatation followed by using the ‘Skeletonization 2D 3D’ function (with the default parameters: Modified Palagyi followed by ‘unsplit shrink’ and distance mapping). The average radius and the area (the sum of the skeleton) were measured using the mean function. Then the ‘Analyze Skeleton (2D/3D)’ function was run to measure and sum the number of junctions.

#### Vessel diameter associated with iPC and iSMC calculation

The 3D cultures were stained for markers for ECs (TJP1 or PECAM1), mural cells (TALGN) or ASs (S100b). The positions of cells touching the vessel-like structures (TJP1 or PECAM1 positive) were determined and the diameters of the vessels closest to these positions were measured using ImageJ software.

### Animal work

Animal experiments were performed in accordance with the German Animal Welfare Law (§4 TschG) and approved by the Government of Upper Bavaria (Vet_02-18-21). Animals were kept under standard conditions at 20–24 °C and 45–65% humidity on a 12-h light-to-dark cycle and had free access to food and water in a pathogen-free facility. All studies were conducted in a mixed gender mice group aged 6 months on a C57BL/6J background. All tissues from the same study were harvested in parallel and during the same time of day.

#### Induction of Foxf2 deletion in adult mice

To induce *Foxf2* deletion, recombination of LoxP sites was induced by 100-µl intraperitoneal injections of 0.25 mg kg^−1^ of tamoxifen (Sigma-Aldrich, cat. no. T5648-5G) in mygliol (Caesar & Loretz, cat. no. 1115805) on 3 d alternately. Brain specimens were obtained from tamoxifen-treated *Foxf2*^*fl/fl*^*;Cdh5-Cre* (*Foxf2*^*iECKO*^) and *Foxf2*^*fl/fl*^ (control) mice at age 6 months.

#### Tissue harvesting

For all molecular studies, mice were first deeply anesthetized using ketamine (100 mg kg^−1^, intraperitoneally), xylazine (10 mg kg^−1^ intraperitoneally). For vessel isolation, mice were transcardially perfused with 20 ml of ice-cold 1× Hank’s balanced salt solution and dissected. After perfusion, the brain was surgically removed from the skull and immediately frozen on dry ice and stored at −80 °C for vessel isolation. For immunohistochemical analysis, anesthetized mice were transcardially perfused with 1× Hank’s balanced salt solution and fixed with 4% PFA. The dissected brain samples were incubated overnight in 4% PFA, followed by vibratome sectioning. For EM, mice were transcardially perfused with 0.1 M sodium cacodylate buffer and fixed with 4% PFA and 2.5% glutaraldehyde in 0.1 M sodium cacodylate buffer, pH 7.4 (Science Services). After dissection, brains were post-fixed for 24 h by immersion and sectioned coronally using vibratome. Brain slides were incubated overnight in the same fixative and then stored in PBS at 4 °C until the start of the postembedding.

#### Vessel isolation

Brain vessels were isolated from a half forebrain as previously described^70,71^. In brief, tissue was homogenized using a glass tissue grinder (Wheaton) in 15 ml of cold Minimum Essential Medium (Thermo Fisher Scientific, cat. no. 11095080) followed by myelin removal using a 15% Ficoll (Sigma-Aldrich, cat. no. F4375-250G) gradient. Isolated vessels were then pelleted and resuspended in PBS with 1% bovine serum albumin (BSA, fraction V, Sigma-Aldrich, cat. no. 10735096001), transferred on to a 40-µm cell strainer (Corning, cat. no. 431750) and extensively washed with 250 ml of cold PBS. Isolated vessels were collected by inverting the cell strainer and washing with cold PBS into a centrifuge tube, followed by centrifugation at 3,000*g* for 5 min.

### Immunofluorescence

IDs of primary and secondary antibodies and dilutions used for all experiments are specified in Supplementary Table 6.

#### Isolated vessels

After isolation, vessels were transferred immediately onto a microscope slide and dried at RT. Slices were kept at 4 °C until staining started. Vessels were fixed using ice-cold 100% acetone for 10 min at −20 °C. Before antibody incubation, vessels were blocked for 1 h at RT using 1% BSA solution in PBS. Primary antibodies were diluted in the same blocking buffer and incubated overnight at 4 °C. Secondary antibodies were diluted in PBS and incubated for 1 h at RT. After washing, DNA was stained using DAPI 1:2,000 solution in PBS for 5 min at RT. Isolated vessels were mounted using Fluoromount medium.

#### The iPS cells, iPS-cell-derived and primary cells

Before staining, iPS cells and iPS-cell-derived cells were seeded into coated coverslips (Marienfeld, cat. no. 0107052) using their corresponding coating. When cells reached confluency, they were fixed using 4% PFA for 15 min at RT or with ice-cold 100% methanol for 15 min at −20 °C. Cells were permeabilized using 0.1% Triton X-100 for 5 min at RT and blocked using 1% BSA solution in PBS for 1 h at RT before antibody incubation. Primary antibody was diluted in the same blocking buffer and incubated at 4 °C overnight. Secondary antibody was diluted in PBS and incubated for 2 h at RT. After washing, DNA was stained using DAPI 1:2,000 solution in PBS for 5 min at RT. Coverslips containing the cells were mounted using Fluoromount medium.

#### The 3D in vitro BBB model

Cells in the 3D microfluidic chambers were fixed using 4% PFA for 15 min at RT as described for feeding. Cells were permeabilized using 0.1% Triton X-100 in MiliQ H_2_O for 5 min at RT. Next, cells were blocked using 1% BSA solution in PBS for 30 min at RT before antibody incubation. The primary antibody was diluted in blocking buffer, whereas the secondary antibody was diluted in PBS. Both antibodies were incubated overnight at 4 °C. DNA was stained using DAPI 1:2,000 solution in PBS for 5 min at RT. Microfluidic chambers were imaged immediately.

### Confocal microscopy and image analysis

Fluorescent images were acquired with either a Zeiss LSM880 or a Zeiss LSM980 confocal microscope using ×10, ×40 and ×64 objectives on ZEN blue or black software. All images were aquired as *z*-stacks of 0.5-μm slices. Images were further processed and analyzed using ImageJ software: for image quantification, integrated density was determined after thresholding. All images shown are representative maximum-intensity projections of the acquired *z*-stacks.

### Electron microscopy

#### TEM of human cell culture

For the ultrastructural analysis of human cells in culture we positioned freshly plasma-coated ACLAR (plastic) films (Science Services) into the cell culture dish before seeding. Then, 5% glutaraldehyde (EM-grade, Science Services) in 0.2 M cacodylate buffer, pH 7.4 (Science Services), prewarmed to 37 °C, was added 1:1 to the cell culture medium and replaced by 2.5% glutaraldehyde in 0.1 M cacodylate buffer after 5 min. Dishes were incubated for a further 25 min on ice. Cells were washed 3× for 5 min with 0.1 M cacodylate buffer on ice and stored in buffer at 4 °C until postfixation in reduced osmium (1% osmium tetroxide (Science Services) and 0.8% potassium ferrocyanide (Sigma-Aldrich) in 0.1 M sodium cacodylate buffer). After contrasting in aqueous 0.5% uranyl acetate (Science Services), the cell monolayer was dehydrated in an ascending ethanol series, infiltrated in Epon (Serva) and cured for 48 h at 60 °C. Blocks were trimmed (TRIM2, Leica) and 50-nm to 80-nm ultrathin sections were generated on an ultramicrotome (UC7, Leica) and deposited on formvar-coated copper grids (Plano) without postcontrasting. TEM micrographs were acquired on a JEM 1400plus (JEOL) equipped with a XF416 camera (TVIPS) and the EM-Menu software (5.2.33, TVIPS). Image analysis was performed using Fiji^72^.

#### Ultrastructural analysis of human 3D cultures

Fixation and heavy metal staining of cells in microfluidic chambers was performed by the application of a drop of reagent on a channel opening and removal at the opposite side. Reagent exchange was performed for 5 min and then at regular intervals for the incubation times mentioned in the following protocol. Cultures in microfluidic chambers were fixed in 2.5% glutaraldehyde in 0.1 M cacodylate for 15 min at 37 °C and 45 min on ice. Heavy metal staining was accomplished by 1 h of incubation in reduced (2.5% potassium ferricyanide) osmium tetroxide in 0.1 M sodium cacodylate on ice. Washes in buffer and water were followed by 1% uranyl acetate in water at 4 °C overnight and 40 °C for 2 h before washes in buffer. All dehydration steps in an ethanol series (at 10% intervals) were performed on ice, followed by RT treatments with 100% ethanol and 100% acetone. Infiltration was performed at 25% and 50% LX112 resin (LADD) in acetone. The plastic from the bottom of the microfluidic chamber was removed for further resin infiltration at 75% and 3× 100% LX112 for 20 min. Fresh resin was added to the opened chamber overnight and for a further 2 h before polymerization at 60 °C for 2 d. Resin-bearing cells were removed from the chamber and mounted on empty resin blocks. The blocks were trimmed, sectioned and imaged by SEM as described for mouse tissue. Adjacent sections were collected on to copper grids for TEM investigation as described for 2D cell cultures. This allowed an overview imaging of regions of interest by SEM and consecutive high-resolution acquisition at TEM.

#### SEM of mouse tissue

For ultrastructural analysis of vessel cross-sections, mice were perfused with 4% PFA and 2 mM calcium chloride in 1× PBS, pH 7.4 (Science Services). Only one hemisphere was used for ultrastructural analysis and was therefore further fixed by immersion in 4% PFA, 2.5% glutaraldehyde and 2 mM calcium chloride in 0.1 M cacodylate buffer for 24 h. Fixed brains were sectioned using a vibratome, further incubated with the same fixative for 24 h and stored in 0.1 M cacodylate buffer at 4 °C until the start of the postembedding.

We applied a rOTO en bloc staining protocol including postfixation in 2% osmium tetroxide (EMS), 1.5% potassium ferricyanide (Sigma-Aldrich) in 0.1 M sodium cacodylate (Science Services) buffer (pH 7.4)^73^. The staining was further enhanced by incubation with 1% thiocarbohydrazide (Sigma-Aldrich) for 45 min at 40 °C. After washing with water, the tissue was incubated in 2% aqueous osmium tetroxide, washed and further contrasted by overnight incubation in 1% aqueous uranyl acetate at 4 °C and 2 h at 50 °C. Dehydration using ascending ethanol series and infiltration with LX112 (LADD) was further carried out on the samples. The final blocks were cured and trimmed (TRIM2, Leica).

For ultrastructure analysis, using a 35° ultra-diamond knife (Diatome) on a ultramicrotome (UC7, Leica) 100-nm-thick sections were taken and collected on to either 1 × 0.5 cm^2^ carbon nanotube tape strips (Science Services) or TEM grids as described. Samples were then attached to adhesive carbon tape (Science Services) on 4-inch silicon wafers (Siegert Wafer) and grounded by adhesive carbon tape strips (Science Services). EM micrographs were acquired using a Crossbeam Gemini 340 SEM (Zeiss) with a four-quadrant backscatter detector at 8 kV using ATLAS5 Array Tomography (Fibics). Medium lateral resolution images (40–100 nm) allowed the identification of blood vessels that were in turn reimaged at 4-nm resolution. Higher-resolution imaging of sections on grids was performed using a JEM 1400plus (JEOL) as described^72^.

### RNA analysis

#### RNA extraction

RNA was extracted from cell pellets or half-cerebellum using TRIzol (QIAGEN, cat. no. 79306) and purified using the RNeasy mini kit (QIAGEN, cat. no. 74106) following the manufacturer’s instructions. Total RNA concentration was determined using a NanoDrop spectrophotometer. RNA was stored at −80 °C.

#### Complementary DNA synthesis

Complementary DNA synthesis was performed immediately after RNA isolation to avoid freezing and thawing cycles. The cDNA was synthesized from 250 ng to 1 µg of RNA using the Omniscript RT kit (QIAGEN, cat. no. 205113) following the manufacturer’s instructions. The cDNA was stored at −20 °C until use.

#### RT-qPCR

Real-time qPCR (RT-qPCR) was performed using SYBR Green Master Mix (QIAGEN, cat. no. 208056) and reactions were set according to the manufacturer’s instructions. Detection was done using a Roche thermocycler. Primer sequences are listed in Supplementary Table 6.

### Protein extraction

#### The iPS cells, iPS-cell-derived somatic cells and primary cells

Protein was lysed in a buffer containing 100 mM Tris-HCl, pH 7.6 (Roth, cat. no. 9090.3), 4% sodium dodecylsulfate (Serva, cat. no. 20765.03) and 100 mM dithiothreitol (Sigma-Aldrich, cat. no. 3483-12-3) by homogenization with a Dounce tissue grinder (Wheaton) and heating for 3 min at 95 °C. Samples were further sonicated (30 s, amplitude 100%, duty cycle 50%) 5× with intermediate cooling using a VialTweeter sonicator (Hielscher). Protein lysates were centrifuged at 16,000*g* for 15 min at 4 °C for removal of undissolved material. The supernatant was transferred to a new tube and stored at −20 °C until further analysis.

#### The iECs (WT versus FOXF2^KO^)

Protein was extracted from iECs using radioimmunoprecipitation buffer containing 150 mM NaCl (Roth, cat. no. 3957.1), 1 M Tris-HCl, pH 7.5 (Roth, cat. no. 9090.3), 1% NP40 (Sigma-Alrich, cat. no. 74385), 0.5 deoxycholate (Roth, cat. no. 3484.3), 0.1% sodium dodecylsulfate and protein inhibitor cocktail (Roche, cat. no. 4693159001). Cell pellets were resuspended in 100 µl of radioimmunoprecipitation buffer and incubated on ice for 30 min. The protein suspension was obtained from the supernatant after centrifugation at 18,000*g* for 30 min at 4 °C and kept at −20 °C for further analysis.

### MS and data analysis

#### Sample preparation

Samples in SDT buffer (15–20 µg of protein) were diluted with water to 50 µl and sonicated with a M-220 focused-ultrasonicater (Covaris) to disrupt DNA and RNA. Afterwards, samples were subjected to proteolytical digestion using a slightly modified single-pot, solid-phase, enhanced sample preparation (SP3) method^74^. Briefly, proteins were bound to 200 µg of a 1:1 mixture of hydrophilic and hydrophobic magnetic Sera-Mag SpeedBeads (GE Healthcare) using a final concentration of 70% (v:v) acetonitrile at 1,200 rpm on a thermomixer (Eppendorf) for 30 min at RT. Beads were retained on a Dynamag-2 (Thermo Fisher Scientific) magnet and the solvent was removed. Cysteine residues were alkylated by the addition of 40 mM iodoacetamide (Sigma-Aldrich) in 50 mM ammonium bicarbonate for 30 min at RT in the dark. Afterwards, the reaction was quenched by adding dithiothreitol to a final concentration of 40 mM. Then, proteins were bound again to the beads, adding acetonitrile to a final concentration of 70% (v:v) for 30 min while shaking. Beads were washed 4× with 200 µl of 80% (v:v) ethanol. For proteolytic digestion, LysC (Promega) was added to 20 µl of 50 mM ammonium bicarbonate with a protease-to-protein ratio of 1:80 and added to the beads. Samples were incubated on a Thermomixer (Eppendorf) for 30 min at 1,000 rpm and 37 °C. Afterwards, trypsin (Promega) was added in 20 µl of 50 mM ammonium bicarbonate with a protease-to-protein ratio of 1:80, followed by an incubation for 16 h at RT.

The second cell-type comparison experiment was processed with the auto-SP3 protocol on a Bravo pipetting robot as previously described^75^. Beads were retained with a magnetic rack and the supernatants were collected. Next, 20 µl of 0.1% formic acid was added to the magnetic beads, followed by sonication for 30 s in a sonication bath (Hielscher Ultrasonics GmbH). The supernatants of each sample were combined, filtered with 0.22-µm spin filters (Costar Spin-x, Corning) to remove remaining beads and dried by vacuum centrifugation. Dried peptides were dissolved in 20 µl of 0.1% formic acid. The peptide concentration after proteolytic digestion was estimated using the Qubit protein assay (Thermo Fisher Scientific).

#### Mass spectrometry

Samples were analyzed in random order on a NanoElute nano-HPLC coupled online with a captive spray ion source to a TimsTOF pro-mass spectrometer (Bruker). An amount of 350 ng of peptides was separated on an in-house packed C18 analytical column (15 cm × 75 µm inner diameter, ReproSil-Pur 120 C18-AQ, 1.9 µm, Dr. Maisch GmbH) using a binary gradient of water and acetonitrile (buffer B) containing 0.1% formic acid at a flow rate of 300 nl min^−1^ (0 min, 2% B; 2 min, 5% B; 70 min, 24% B; 85 min, 35% B; 90 min, 60% B; or 0 min, 2% B; 2 min, 5% B; 62 min, 24% B; 72 min, 35% B; 75 min, 60% B) and a column temperature of 50 °C. Data Independent Acquisition Parallel Accumulation–Serial Fragmentation (DIA-PASEF) methods with a cycle time of 1.8 s (cell-line comparison) and 1.4 s (FOXF2^KO^ versus WT iECs, cell-line comparison 2) were used for spectrum acquisition. Briefly, ion accumulation and separation using trapped ion mobility spectrometry (TIMS) was set to a ramp time of 100 ms. One scan cycle included one TIMS full MS scan and the DIA-PASEF windows covered the *m*/*z* range from 350 *m*/*z* to 1,200 *m*/*z* (1.8-s method) or from 350 *m*/*z* to 1,000 *m*/*z* (1.4-s method) with 34 or 26 windows of 27 *m*/*z* with an overlap of 1 *m*/*z*, respectively.

#### MS raw data analysis

The raw data were analyzed using DIA-NN software (v1.8 or v2.0.2)^76^ for protein label-free quantification (LFQ). A one-protein-per-gene canonical fasta databases of *Homo* *sapiens* (download: 1 March 2023, 20,603 entries for FOXF2 KO versus WT iECs and 9 December 2024, 20,662 entries for the cell-line experiments) from UniProt and a fasta database with 246 common potential contaminations from Maxquant were used to generate the spectral libraries with DIA-NN, which include 12,813, 9,682 and 12,511 proteins, respectively. Trypsin was defined as a protease. Two missed cleavages were allowed and peptide charge states were set to 2–4. Carbamidomethylation of cysteine was defined as a static modification. Acetylation of the protein amino-terminus as well as oxidation of methionine were set as variable modifications. The FDR for both peptides and proteins was adjusted to <1%. Two peptides were required for protein LFQ for all datasets in the manuscript.

#### Comparison with published datasets

In addition to our own proteomics data, we incorporated data (accession no. PXD029380 using only control samples) from ref. ^31^ and the brain vascular proteome and whole-brain proteome datasets (https://www.synapse.org/Synapse:syn52559738, using only amyloid-negative control samples) from ref. ^32^. We focused our analysis on specific markers for ECs, SMCs, pericytes and ASs, which we defined by selecting the top 100 genes curated from the ref. ^33^ dataset. Datasets were merged by gene symbol and genes with more than three missing values were excluded. Heatmaps were generated using the pheatmap package (1.0.12) in R, with rows hierarchically clustered and columns arranged by dataset. For the astrocyte analysis, only the Wojtas ‘whole-brain’ dataset was used, whereas the vascular dataset was employed for the other cell types.

#### PCA

Proteomic data of primary and induced human cells were broken down into four components using the python package sklearn (v1.1.2) after standardization (removing the mean and scaling to unit variance) with the StandardScaler function of the same package. Data were imputed using zero imputation.

#### Enrichment analysis

Enrichment analysis of biological processes (GOTERM_BP_DIRECT) was performed with Database for Annotation, Visualization and Integrated Discovery (DAVID) software (v6.8)^77,78^ (https://david.ncifcrf.gov/home.jsp) using *Homo sapiens* standard background dataset. For PCA-based enrichment analysis iPS cells were compared to iPS-cell-derived cells by PCA and the top 250 proteins defining PC1 were used for enrichment analysis. For iPS-cell-derived cell characterization, significant proteins (Student’s *t*-test, *P* < 0.05) with minimum ± fivefold change were used for enrichment analysis. For FOXF2^KO^ iEC analysis, all significant proteins (Student’s *t*-test, *P* < 0.05) were used for enrichment analysis.

#### Correlation analysis

Correlation analysis was performed using linear regression on GraphPad Prism (v10.4). All common quantified proteins in a minimum of three samples were used for the ‘all proteins’ correlation. Cell-type lineage-specific proteins were filtered for the ‘specific proteins’ correlation. Cell-specific lineage was performed by filtering using GO terms and cell-specific markers obtained from PanglaoDB (https://panglaodb.se/), setting up the filters for ‘human samples’. Further filtering was done using the following GO terms: endothelial cell differentiation, endothelial cell development, endothelial cell proliferation, endothelial cell activation, endothelial cell–cell adhesion, endothelial cell–matrix adhesion, astrocyte development, astrocyte differentiation, astrocyte activation, astrocyte migration, astrocyte projection, smooth muscle cell differentiation, smooth muscle cell contraction, smooth muscle cell proliferation, smooth muscle cell migration and pericyte differentiation.

### Bulk RNA-seq

#### Library preparation and sequencing

Library construction was done as previously described by BGI^79^ using 400 ng of RNA of each cell type. Bwa-mem2 (v2.2)^80^ was used for aligning the reads with the human genome of reference GRCh38. Normalization was performed using edgeR (v4.4.0)^81^.

#### Comparison with published datasets

FASTQ files were processed using soapnuke (v2.1.8)^79^, including adapter and low-quality trimming^78^ and quality-controlled reads were subsequently aligned to the GRCh38 reference genome using STAR v2.7.11b (ref. ^82^). Count tables for each sample were generated using STAR’s ‘quantMode’ option. To compare our bulk RNA-seq data with published human cerebrovasculature datasets, we obtained data from ref. ^34^ via accession no. GSE256493 and from ref. ^33^ via accession no. GSE163577, restricting both to nondiseased control samples. Single-cell datasets were imported as Seurat objects and pseudo-bulk data for each cell type were produced using the AggregateExpression function. Raw counts were filtered and trimmed mean of M-values (TMM) normalized with edgeR^81^; the resulting log_2_(transformed counts per million) values were analyzed via PCA using the prcomp function in R v4.4.2. EC subtypes (artery, capillary, vein) from both datasets were grouped together under ‘Endothelial cells’ for simplification. PCA plots were visualized with the factoextra package. PCs 1–4 were compared to determine the most meaningful data separation. As PC1–PC2 and PC2–PC3 separated the samples based on experimental groups (suggesting a batch effect of the analysis), PC3–PC4 was chosen for representation. All plots are available in Source data for Extended Data Fig. 6d.

### Statistical analysis and reproducibility

Data collection and analysis were performed without blinding of the experimental groups. LFQ intensities of the proteomics data were log_2_(transformed) before statistical testing. Proteomic, transcriptomic and morphological datasets showed normal distribution (tested with GraphPad Prism (v10.4)). Statistical significance was analyzed using GraphPad Prism except for proteomics analysis where Perseus (v1.6.2.3) and Excel (v16.9) were used. Significance was analyzed by Student’s *t*-test (two tailed, unpaired) when two groups were analyzed or by ANOVA when more than two groups were analyzed. Perseus was used to apply a permutation-based FDR estimation for multiple hypotheses^83^. Multiple comparisons were corrected as recommended by GraphPad using Tukey’s method. All graphs show mean ± s.d. unless stated otherwise. The *P-*value significance follows criteria recommended by GraphPad: ^*^P < 0.05, ^**^P < 0.01, ^***^P < 0.001 and ^****^P < 0.0001. Statistical details are also specified in the figure legends. All experiments were repeated at least three times, including imaging. No statistical methods were used to predetermine sample sizes, but sample sizes were determined according to those reported in previous publications^71,84^. Samples in MS were measured in random order. The other experiments did not require randomization because samples were homogeneous with respect to key characteristics (such as genetic background, sex and age) and controls were isogenic. Blinding was applied to in vivo experiments, tissue processing, microscopy (in vivo and in vitro) and image analysis (in vivo and in vitro).

### Reporting summary

Further information on research design is available in the Nature Portfolio Reporting Summary linked to this article.

## Online content

Any methods, additional references, Nature Portfolio reporting summaries, source data, extended data, supplementary information, acknowledgements, peer review information; details of author contributions and competing interests; and statements of data and code availability are available at 10.1038/s41593-025-02123-w.

## Supplementary information

Reporting Summary

Supplementary Video 1 Flow of human blood in iPSC model.

Supplementary Video 2 Fluo4 signal in iPSC BBB model after Ca2+ addition.

Supplementary Table 1 All proteins quantified in iPS-cell-derived (iECs, iPCs, iSMCs and iASs) and primary cells (pECs, HUVECs, pPCs, pSMCs, pASs). The sheet labeled ‘information’ contains a summary of all abbreviations used in Supplementary Table 1. Columns E–N, X–Z, AF, AK and AP, F–BC indicate average of protein abundance in each cell type (*n* = 4–5 biological replicates per group for iPSC-derived cells and 4–5 technical replicates per group for primary cells). Columns BO–GJ indicate LFQ log_2_(values) obtained during MS quantification.

Supplementary Table 2 All proteins quantified in iPS-cell-derived cells (iECs, iPCs, iSMCs and iASs) compared to iPS cells. The sheet labeled ‘information’ contains a summary of all used abbreviations in further sheets. Column E indicates protein abundance changes in each specific cell type versus iPSCs. Column F indicates *P* value and the red color denotes statistical significance (*n* = 5 replicates per group, Student’s *t*-test *P* < 0.05).

Supplementary Table 3 Summary of endothelial cell TEER values in different studies.

Supplementary Table 4 All quantified mRNAs from bulk RNA-seq of iECs, pECs, HUVECs, iPCs, pPCs, iSMCs and pSMCs. Column A indicates ENSMEBL gene ID. Columns B–AH indicate counts per million (cpm) of each transcript in each specific cell type.

Supplementary Table 5 All proteins quantified in iEC from WT and FOXF2^KO^ lines. The heet labeled ‘information’ contains a summary of all used abbreviations in Supplementary Table 3. Column F indicates protein abundance changes in iECs WT versus FOXF2^KO^. Column G indicates *P* values, the red color denoting statistical significance (*n* = 5 replicates per group, Student’s *t*-test *P* < 0.05).

Supplementary Table 6 Reagents. Sheets contain LNP formulations, antibodies and primers used for the study.

## Source data

Source data for Figs. 1–5 and Extended Data Figs. 1–3, 6, 7, 9 and 10. All source data for bar graphs.

Source Data Extended Data Fig. 6 PCA based on all transcripts detected in iECs, iPCs and iSMCs compared to published datasets^33,34^. EC subtypes preset in the published data have been grouped into one general EC group for simplification. Comparisons of PC1–PC2, PC2–PC3 and PC3–PC4 are shown.

## Data Availability

The proteomics data have been deposited to the ProteomeXchange Consortium via the PRIDE^79^ partner repository with accession nos. PXD051959, PXD051960 and PXD066414. Bulk RNA-seq dataset are available at the Gene Expression Omnibus with accession no. GSE302761. All data supporting the findings described in this manuscript are available in the article, Supplementary Tables 1–6, our online database and from the corresponding authors upon request. Source data are provided with this paper.
