# Supplementary material for: A fully iPS-cell-derived 3D model of the human blood–brain barrier for exploring neurovascular disease mechanisms and therapeutic interventions
Source: Nat Neurosci. 2025 Dec 15;29(2):479–92. doi: 10.1038/s41593-025-02123-w (PMC12880921; doi:10.1038/s41593-025-02123-w)
Supplement: Supplementary file 1 — Reporting Summary [file 41593_2025_2123_MOESM1_ESM.pdf]

nature portfolio

Corresponding author(s): Dominik Paquet
Martin Dichgans

Last updated by author(s): 01.08.2025

Reporting Summary

Nature Portfolio wishes to improve the reproducibility of the work that we publish. This form provides structure for consistency and transparency in reporting. For further information on Nature Portfolio policies, see our Editorial Policies and the Editorial Policy Checklist.

Statistics

For all statistical analyses, confirm that the following items are present in the figure legend, table legend, main text, or Methods section.

n/a	Confirmed	
☐	☒	The exact sample size (*n*) for each experimental group/condition, given as a discrete number and unit of measurement
☐	☒	A statement on whether measurements were taken from distinct samples or whether the same sample was measured repeatedly
☐	☒	The statistical test(s) used AND whether they are one- or two-sided<br>*Only common tests should be described solely by name; describe more complex techniques in the Methods section.*
☒	☐	A description of all covariates tested
☐	☒	A description of any assumptions or corrections, such as tests of normality and adjustment for multiple comparisons
☐	☒	A full description of the statistical parameters including central tendency (e.g. means) or other basic estimates (e.g. regression coefficient) AND variation (e.g. standard deviation) or associated estimates of uncertainty (e.g. confidence intervals)
☐	☒	For null hypothesis testing, the test statistic (e.g. *F*, *t*, *r*) with confidence intervals, effect sizes, degrees of freedom and *P* value noted<br>*Give P values as exact values whenever suitable.*
☒	☐	For Bayesian analysis, information on the choice of priors and Markov chain Monte Carlo settings
☒	☐	For hierarchical and complex designs, identification of the appropriate level for tests and full reporting of outcomes
☐	☒	Estimates of effect sizes (e.g. Cohen's *d*, Pearson's *r*), indicating how they were calculated

Our web collection on statistics for biologists contains articles on many of the points above.

Software and code

Policy information about availability of computer code

Data collection	Zeiss Confocal microscope (LSM880 and LSM980) using 25x, 40x and 63x objectives; nanoElute nanoHPLC which was coupled to a TimsTOF pro mass spectrometer with a CaptiveSpray ion source (Bruker, Germany); JEM 1400plus (JEOL) equipped with a XF416 camera (TVIPS)
Data analysis	EM-Menu software (version 5.2.33, TVIPS) for EM image analysis; Maxquant (1.6.17), DIA-NN (1.8 and 2.0.2), Perseus (1.6.2.3), and pheatmap package (1.0.12) for analysis of proteomic data and statistics; ImageJ/Fiji (1.52p and 1.54f) for image processing and quantification; python package sklearn (1.1.2), GraphPad (10.4) and Excel (16.9) for statistics and figure representation; STAR (2.7.11b), Bwa-mem2 (2.2), soapnuke (2.1.8), and edgeR (4.4.0) for analysis of sequencing data; R (version 4.4.2) for comparison with published datasets, statistics and figure representation.

For manuscripts utilizing custom algorithms or software that are central to the research but not yet described in published literature, software must be made available to editors and reviewers. We strongly encourage code deposition in a community repository (e.g. GitHub). See the Nature Portfolio guidelines for submitting code & software for further information.

Data

Policy information about availability of data

All manuscripts must include a data availability statement. This statement should provide the following information, where applicable:

- Accession codes, unique identifiers, or web links for publicly available datasets
- A description of any restrictions on data availability
- For clinical datasets or third party data, please ensure that the statement adheres to our policy

The proteomics data have been deposited to the ProteomeXchange Consortium via the PRIDE84 partner repository with the dataset identifiers PXD051959, PXD051960, and PXD066414. BulkRNA seq dataset are available at GEO with the accession number GSE302761. All data supporting the findings described in this manuscript are available in the article, the supplementary materials, our online database, and from the corresponding authors upon request.

Research involving human participants, their data, or biological material

Policy information about studies with human participants or human data. See also policy information about sex, gender (identity/presentation), and sexual orientation and race, ethnicity and racism.

Reporting on sex and gender	Female human iPSCs line A18944 (ThermoFisher, Cat# A18945)
Reporting on race, ethnicity, or other socially relevant groupings	N/A
Population characteristics	N/A
Recruitment	N/A
Ethics oversight	N/A

Note that full information on the approval of the study protocol must also be provided in the manuscript.

Field-specific reporting

Please select the one below that is the best fit for your research. If you are not sure, read the appropriate sections before making your selection.

☒ Life sciences ☐ Behavioural & social sciences ☐ Ecological, evolutionary & environmental sciences

For a reference copy of the document with all sections, see nature.com/documents/nr-reporting-summary-flat.pdf

Life sciences study design

All studies must disclose on these points even when the disclosure is negative.

Sample size	Sample sizes were determined based on results obtained in previous proteomic and immunohistochemical studies on brain vessels published by the authors (Todorov-Völgyi et al., 2024 and Zellner et al., 2018).
Data exclusions	No data was excluded from the study.
Replication	Animal-based experiments included 4-8 independent animals per genotype and were replicated at least 3 times. iPSC-BBB experiments were replicated at least 3 times. All replication attempts were always sucessful.
Randomization	Samples in mass spec were measured in random order. The other experiments did not require randomization as samples were homogeneous with respect to key characteristics (such as genetic background, sex, age) and controls were isogenic.
Blinding	Blinding was applied to in vivo experiments, tissue processing, microscopy (in vivo and in vitro), image analysis (in vivo and in vitro) and proteomics. Computational analysis did not require blinding, as all samples were analysed in an automated way with the same algorithms.

Reporting for specific materials, systems and methods

We require information from authors about some types of materials, experimental systems and methods used in many studies. Here, indicate whether each material, system or method listed is relevant to your study. If you are not sure if a list item applies to your research, read the appropriate section before selecting a response.

Materials & experimental systems

n/a	Involved in the study
☐	☒ Antibodies
☐	☒ Eukaryotic cell lines
☒	☐ Palaeontology and archaeology
☐	☒ Animals and other organisms
☒	☐ Clinical data
☒	☐ Dual use research of concern
☒	☐ Plants

Methods

n/a	Involved in the study
☒	☐ ChIP-seq
☒	☐ Flow cytometry
☒	☐ MRI-based neuroimaging

Antibodies

Antibodies used

CAV1 Rabbit Cell signaling 3267S 1:250 (3D) 1:500 (2D)
CD248 Rabbit Invitrogen PA5-84090 1:100 (2D, PFA)
CDH5 Goat R&D systems AF938 1:100 (3D) 1:250 (2D, PFA)
CLDN5 Mouse ThermoFischer 35-2500 1:100 (2D, Methanol) 1:100 (3D)
CNN1 Rabbit Millipore 04-589 1:250 (2D, PFA)
COL4 Goat Southern biotech 1340-01 1:250 (2D, PFA) 1:100 (3D)
ERG Rabbit Abcam ab92513 1:100 (2D, PFA)
GFAP Guinea pig Synaptic systems 173 004 1:100 (3D) 1:500 (2D, PFA)
KCNJ8 Mouse Invitrogen MA5-27679 1:100 (2D, PFA)
NANOG Rabbit Cell signaling 4903S 1:500 (2D, PFA)
NG2 Rabbit Millipore AB5320 1:250 (2D, PFA)
NOS3 Rabbit Abcam ab5589 1:150 (2D, PFA)
OCLN Rabbit Invitrogen 71-1500 1:100 (2D, Methanol)
OCT4 Rabbit Tebubio 09-0023 1:500 (2D, PFA)
PDGFRß Goat R&D systems AF1042 1:250 (2D, PFA)
PECAM1 Mouse Santa Cruz sc-376764 1:100 (3D) 1:250 (2D, PFA)
PGP/ABCB1 Rabbit Abcam ab235954 1:100 (2D, Methanol)
PODXL Mouse R&D systems MAB1658 1:250 (2D, PFA) 1:100 (3D)
S100ß Mouse Sigma S2532 1:500 (2D, PFA)
SLC16A1 Rabbit Origene TA321556 1:100 (2D, Methanol)
SLC2A1 Rabbit ThermoFisher MA5-31960 1:100 (2D, Methanol)
SLC3A2/CD98 Mouse ThermoFischer 66883-1-IG 1:100 (2D, Methanol)
SMA Mouse Sigma C6198 1:250 (2D, PFA)
SSEA4 Mouse Abcam ab16297 1:500 (2D, PFA)
TAGLN Rabbit Abcam ab14106 1:200 (2D, PFA)
TEK Goat R&D systems AF762 1:100 (2D, Methanol)
TFRC Rabbit Abcam ab84036 1:100 (2D, PFA)
TJP1 Mouse ThermoFischer 33-9100 1:100 (2D, PFA -3D) 1:250 (mouse)
TRA160 Mouse Millipore MAB4360 1:500 (2D, PFA)
VWF Rabbit Dako A0082 1:100 (2D, PFA)

Alexa-488 Anti-Mouse Jackson Laboratories 715-546-150 1:500
Alexa-488 Anti-Rabbit Jackson Laboratories 711-545-152 1:500
Alexa-488 Anti-guinea pig Abcam ab150185 1:500
Alexa-488 Anti-Rat Jackson Laboratories 712-546-153 1:500
Cy3 Anti-Mouse Jackson Laboratories 715-165-150 1:500
Cy3 Anti-Rabbit Jackson Laboratories 711-165-152 1:500
Cy3 Anti-Goat Jackson Laboratories 705-165-147 1:500
Cy3 Anti-guinea pig Jackson Laboratories 706-165-148 1:500
Alexa-647 Anti-Mouse Jackson Laboratories 715-606-150 1:500
Alexa-647 Anti-Rabbit Jackson Laboratories 711-606-152 1:500
Alexa-647 Anti-Goat Jackson Laboratories 705-606-147 1:500

Validation

All antibodies have been validated for this application by the supplier as indicated on the websites and datasheets of the antibodies.

Eukaryotic cell lines

Policy information about cell lines and Sex and Gender in Research

Cell line source(s)

iPSCs - A18944: purchased from Thermofisher (Cat. No. A18945)
pEC - Human Brain Microvascular Endothelial Cells (ScienCell, Catalog No. 1000)
pPC - Human Brain Vascular Pericytes (ScienCell, Catalog No. 1200)
pSMC - Human Brain Vascular Smooth Muscle Cells (ScienCell, Catalog No. 1100)
pAS - Human Astrocytes-midbrain (ScienCell, Catalog No. 1850)

	HUVECS - Human Umbilical Vein Endothelial Cells (ThermoFisher, C0035C)<br>hBMEC - Human cerebral microvascular endothelial cells or blood-brain barrier line (Sigma, Cat# SCC066).
Authentication	Authenticated by manufacturer using STR analysis, morphology, and/or marker stainings.
Mycoplasma contamination	The line was regularly tested and confirmed negative for mycoplasma contamination.
Commonly misidentified lines<br>(See ICLAC register)	No such lines were used.

Animals and other research organisms

Policy information about studies involving animals; ARRIVE guidelines recommended for reporting animal research, and Sex and Gender in Research

Laboratory animals	Mus musculus, C57BL/6J, mixed males and females, 6 month old.
Wild animals	The study did not involve wild animals.
Reporting on sex	Mixed gender
Field-collected samples	No field collected samples were used in the study.
Ethics oversight	Animal experiments were performed in accordance with the German Animal Welfare Law (§4 TschG) and approved by the Government of Upper Bavaria (Vet_02-18-21). No additional ethical approval was required.

Note that full information on the approval of the study protocol must also be provided in the manuscript.

Plants

Seed stocks	*Report on the source of all seed stocks or other plant material used. If applicable, state the seed stock centre and catalogue number. If plant specimens were collected from the field, describe the collection location, date and sampling procedures.*
Novel plant genotypes	*Describe the methods by which all novel plant genotypes were produced. This includes those generated by transgenic approaches, gene editing, chemical/radiation-based mutagenesis and hybridization. For transgenic lines, describe the transformation method, the number of independent lines analyzed and the generation upon which experiments were performed. For gene-edited lines, describe the editor used, the endogenous sequence targeted for editing, the targeting guide RNA sequence (if applicable) and how the editor was applied.*
Authentication	*Describe any authentication procedures for each seed stock used or novel genotype generated. Describe any experiments used to assess the effect of a mutation and, where applicable, how potential secondary effects (e.g. second site T-DNA insertions, mosiacism, off-target gene editing) were examined.*
