# Supplementary figures and images for: A fully iPS-cell-derived 3D model of the human blood–brain barrier for exploring neurovascular disease mechanisms and therapeutic interventions
Source: Nat Neurosci. 2025 Dec 15;29(2):479–92. doi: 10.1038/s41593-025-02123-w (PMC12880921; doi:10.1038/s41593-025-02123-w)

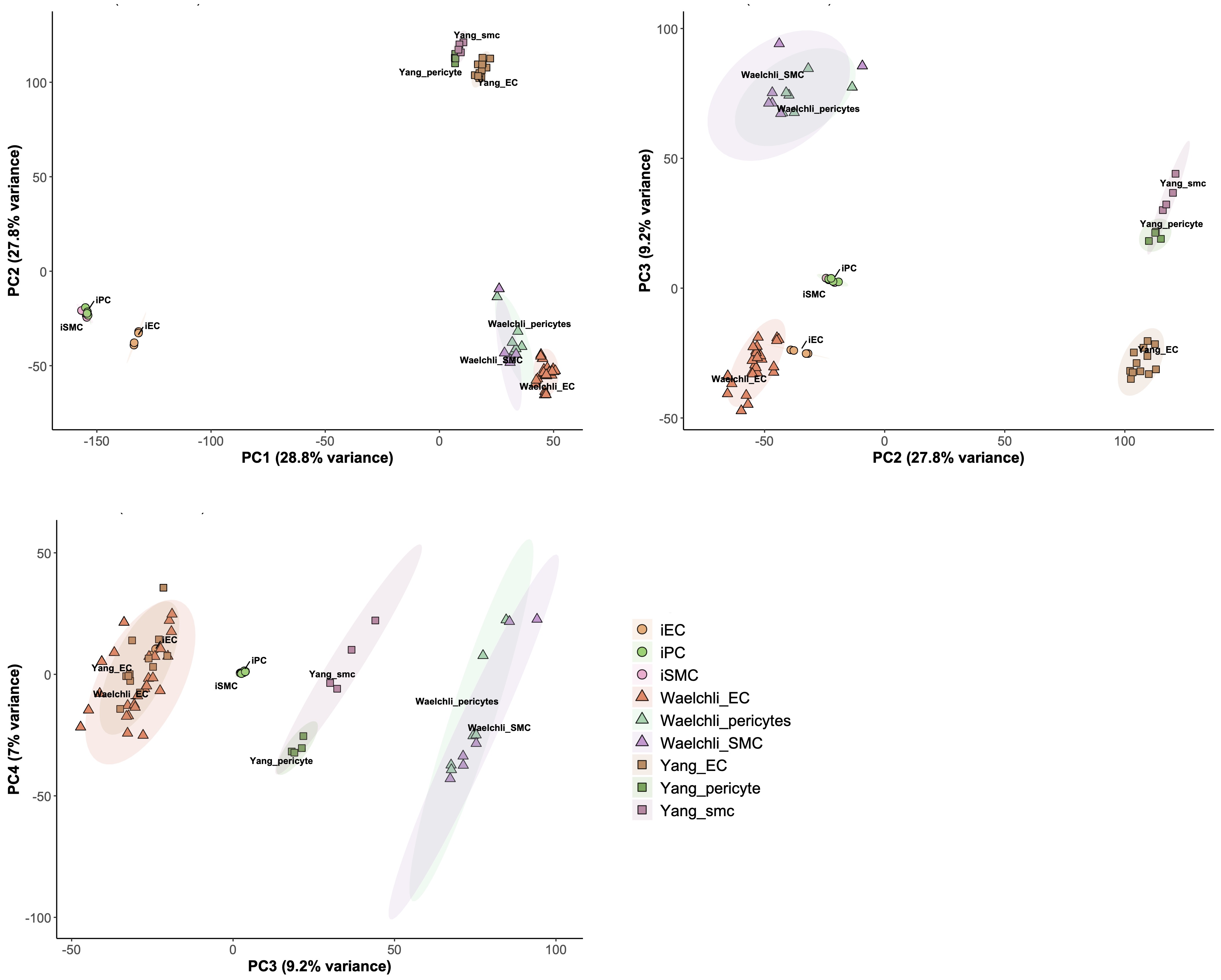

Supplement: Supplementary file 11 — PCA based on all transcripts detected in iECs, iPCs and iSMCs compared to published datasets33,34. EC subtypes preset in the published data have been grouped into one general EC group for simplification. Comparisons of PC1–PC2, PC2–PC3 and PC3–PC4 are shown. [file 41593_2025_2123_MOESM11_ESM.jpg]
